# Adaptive evolution can mitigate the negative effects of temperature stress on plant–pollinator interactions

**DOI:** 10.1111/nph.70705

**Published:** 2025-11-08

**Authors:** Juan Traine, Quint Rusman, Florian P. Schiestl

**Affiliations:** ^1^ Department of Systematic and Evolutionary Botany University of Zürich Zollikerstrasse 107 CH‐8008 Zürich Switzerland

**Keywords:** bumblebees, climate change, floral signals, phenotypic plasticity and trait evolution, plasticity first, pollination, thermomorphogenesis

## Abstract

- Temperature stress negatively affects various aspects of plant fitness, including plant–pollinator interactions, but whether plants can overcome these adverse effects through adaptive evolution is largely unknown.
- Here, we conducted a six‐generation evolution experiment using fast‐cycling *Brassica rapa* plants at ambient and elevated temperatures, with bumblebee pollination. At the end of the experiment, we re‐grew the evolved genotypes at different temperatures. We phenotyped the plants and conducted pollinator bioassays to assess adaptive evolution, evolutionary trait divergence, and the evolution of heat‐mediated phenotypic plasticity.
- We found that plants that had evolved with bumblebee pollination in both temperature regimes had higher seed set than control plants, which suffered lower seed set when evolved under elevated temperatures. We also showed that the number of flowers, a trait that largely determined plant attractiveness to bumblebees and seed set, was increased in bumblebee‐pollinated plants, and so was heat‐induced phenotypic plasticity in flower number. Plants that evolved with high temperature showed increased UV reflection, a stronger association between flower size and nectar content (honest signaling), and reduced scent emission.
- Our results show that plants that evolve under pollinator‐mediated selection can mitigate at least some of the negative effects of temperature stress through adaptive evolution.

## Introduction

Rapid environmental shifts driven by climate change, such as the increase in average temperatures and the number of extreme temperature events, may lead to temperature stress and thus reduced fitness in many organisms, as well as altered patterns of selection and potentially evolutionary change (Anderson *et al*., 2012; Klasen *et al*., 2014; Mitchell & Whitney, 2018; Anderson & Song, 2020; Fischer *et al*., 2022). Indeed, warming can influence plant selective landscapes both through direct temperature effects on plant physiology and by changing how plants interact with other organisms around them (Gilman *et al*., 2012; Scaven & Rafferty, 2013; Alexander *et al*., 2015; Becklin *et al*., 2016; Garcia *et al*., 2023; Nomoto *et al*., 2024). Such heat‐mediated changes in selection may lead to adaptive evolution (Davis *et al*., 2005; Martin *et al*., 2023); for example, several studies have shown that warmer temperatures lead to increased herbivory and reduced pollination success, leading to increased defenses, altered flower trait evolution, and flowering time (Hamann *et al*., 2021a,b).

When plant populations evolve in response to temperature and insect‐mediated changes in selection, this may lead to local adaptation (VanWallendael *et al*., 2019; Lortie & Hierro, 2022). Although there is little evidence of plant local adaptation to different temperatures specifically, there is ample evidence of plant local adaptation to different climates (Lortie & Hierro, 2022). For instance, the North American invasive plant *Ambrosia artemissifolia* showed rapid local adaptation to the climates of its introduced range, in Europe and Australia (van Boheemen *et al*., 2019). Adaptation to a hotter environment may involve increased investment in thermal tolerance mechanisms, which can trade off with other functions (e.g. growth), making them advantageous in a warm temperature range but too costly outside of it (Maher *et al*., 2019; Willi & Van Buskirk, 2022). Heat‐mediated effects on plant–insect interactions may also lead to local adaptation through the evolution of diverging phenotypes, best suited to maximize pollinator attraction and minimize herbivore damage at their local temperature (Briscoe Runquist *et al*., 2020; López‐Goldar & Agrawal, 2021; Dorey *et al*., 2024). Nevertheless, how such abiotic and biotic factors can interactively lead to plant local adaptation remains an understudied topic (Frachon *et al*., 2023; Izquierdo *et al*., 2023).

Plants can respond to temperature increases through phenotypic plasticity, the ability of a genotype to display different phenotypes according to the environment (Schlichting, 1986; Nicotra *et al*., 2010; Casal & Balasubramanian, 2019). The difference between the phenotypes in response to different environments is called ‘reaction norm’. Plants display a comprehensive array of plastic changes in response to temperature increases (also called ‘thermomorphogenesis’; Lee *et al*., 2020), such as leaf and petiole elongation, flower size reduction, and changes in volatile emissions (Gouinguené & Turlings, 2002; Quint *et al*., 2016; Wiszniewski *et al*., 2022; Cordeiro & Dötterl, 2023a). Heat‐induced plasticity can have diverse evolutionary effects, although this connection is little explored. Plasticity can change pollinator‐mediated phenotypic selection (Dorey & Schiestl, 2022) and thus impact adaptive evolution. Reduced flower size and color reflectance (Traine *et al*., 2024), reduced scent emission (Cordeiro & Dötterl, 2023a; Rusman *et al*., 2025) and reduced nectar content (Descamps *et al*., 2021a) have been documented in response to warmer temperatures and may decrease the attractiveness of flowers to pollinators, and so change patterns of selection mediated by pollinators (J. Traine, F. P. Schiestl and Q. Rusman, unpublished). Since plasticity can be a heritable trait itself, it may also undergo evolutionary change, through an evolutionary increase or decrease of the reaction norm (Van Kleunen & Fischer, 2005; Kelly, 2019). If phenotypic plasticity produces phenotypes that are closer to the fitness optimum (e.g. via improving survival and pollination success at high temperatures), then reaction norms might be under selection to be maintained or increased (Givnish, 2002). The plasticity‐first hypothesis links plasticity to trait evolution by stating that plasticity precedes or even enables adaptive evolution (Levis & Pfennig, 2016). But as of yet, no experimental evolution study has tested the interactive effects of temperature and biotic interactions on the evolution of heat‐mediated plasticity, and whether increased plasticity is associated with trait evolution. This topic is important because plasticity may help plants cope with temperature stress, while it may trade off with pollinator attraction and thus be selected against in outcrossing plants.

Experimental evolution is a powerful tool for studying the process of adaptation in real time. Few experimental evolution studies have used plants as model organisms (Schiestl, 2024), but several of those that did have used fast‐cycling *Brassica rapa* plants. Using this outcrossing and thus pollinator‐dependent model, divergent evolution driven by different pollinators, the trade‐off between pollination and herbivory, and adaptation to soil and biotic interactions have been demonstrated (Gervasi & Schiestl, 2017; Ramos & Schiestl, 2019; Dorey *et al*., 2024). Plasticity in response to temperature was also shown earlier in a glasshouse experiment (Traine *et al*., 2024). Here, we investigated whether increased temperature impacts adaptive evolution in traits important for pollination, as well as the reaction norm of these traits in a plant pollinated by bumblebees. We performed a six‐generation evolution experiment using fast‐cycling *B. rapa* plants pollinated by bumblebees, as well as a hand‐pollinated control group. We conducted the experiment in two glasshouses, one with ‘ambient’ temperature (23°C) and one with ‘hot’ temperature (27°C, plus 30°C for 24 h once per week and in total six times during the growth period). We measured phenotypes of the first generation of plants undergoing evolution to assess how warming and biotic interactions (pollination by bumblebees, and in addition also butterflies and a generalized treatment, bumblebees and butterflies) influence natural selection on flower traits (J. Traine, F. P. Schiestl and Q. Rusman, unpublished). At the end of the evolution experiment, we re‐grew the ensuing plant genotypes both at their ‘local’ and ‘foreign’ temperatures, to assess the plasticity and evolutionary change in plant traits and seed set as an indication of adaptive evolution. We focused on traits that are known to be important in plant–pollinator interactions in this system (Schiestl *et al*., 2018; Knauer *et al*., 2021; Traine *et al*., 2024) such as the number and size of flowers, flower signals, nectar, the association between signals and nectar (honest signaling) as well as other traits such as plant height, number of leaves and flowering time. Honest signaling was previously detected for the floral scent compound phenylacetaldehyde and shown to be important for bumblebees to find the most profitable flowers (Knauer & Schiestl, 2015).

In this experimental study, we asked the following specific questions: How do temperature and bumblebee pollination influence the evolution of (1) local adaptation (assessed by fitness and pollinator visitation), (2) plant flowering traits, (3) heat‐mediated plasticity in plant flowering traits, and (4) the association between flower signals and reward (honest signaling). We expected that plants would evolve local adaptation to increased temperature, and thus adaptively reduce the negative effects of heat stress. We also expected that temperature would impact how traits evolve under pollinator‐mediated selection (Rusman *et al*., 2025, since heat‐mediated plasticity reduces some but increases other pollinator advertisement traits in this system (Traine *et al*., 2024). Selection analyses in the starting population of the experiment showed that plants in the hot temperature were under positive selection for flower size but not under selection for flower number, while plants in the ambient temperature were under positive selection for flower number, but not for flower size (J. Traine, F. P. Schiestl and Q. Rusman, unpublished). Hence, we expected that pollinator‐mediated selection may lead to increased flower size but not necessarily more flowers. We expect that adaptive plasticity, that is, plasticity that increases not only the survival of plants in hot conditions but also their attractiveness to pollinators, will increase, whereas plasticity that acts against pollinator attractiveness may remain unchanged or decrease. Indeed, selection analyses on temperature‐induced floral plasticity in the starting population of the experiment showed negative selection on plasticity in flower size (J. Traine, F. P. Schiestl and Q. Rusman, unpublished). Finally, because heat influences floral advertisement and reward evolution as well as plastic trait expression, we expect temperature to influence honest signaling. Because heat‐induced plasticity reduces floral scent emission, bumblebees may switch to use visual signals more than chemical signals (scent), leading to selection/evolution of a stronger association of nectar and visual signals and honest signaling thus becoming more visual.

## Materials and Methods

### Plants and insects

For our evolution experiment, we used fast‐cycling *Brassica rapa* L. plants (Wisconsin Fast Plant, Carolina Biological Supply, Burlington), harboring sufficient genetic variance for rapid evolutionary response (Gervasi & Schiestl, 2017; Ramos & Schiestl, 2019; Dorey *et al*., 2024; Dorey & Schiestl, 2024). These plants are annual and mostly self‐incompatible. Their wild conspecifics are pollinated by a diverse community, which includes buff‐tailed bumblebees (*Bombus terrestris*) (Atmowidi *et al*., 2007; Rader *et al*., 2009). To control for genotype in the starting population, we grew 300 plants from seeds, grouped them in random pairs and cross‐pollinated them by hand, creating full‐sib families (groups of seeds that shared both parents). We made 150 families this way, out of which 98 were used for experimental evolution.

As a pollinator, we used buff‐tailed bumblebees (*B. terrestris*). This generalist pollinator species is a common visitor and efficient pollinator of plants in the genus *Brassica* (Rader *et al*., 2013; Frachon *et al*., 2023). Plus, selection by *B. terrestris* leads to rapid evolution in *B. rapa* plants (Gervasi & Schiestl, 2017; Ramos & Schiestl, 2019). Bumblebee hives were purchased from Andermatt Biocontrol (Grossdietwil, Switzerland); two hives were purchased per generation and used for each bioassay to control for hive‐specific effects. Hives were kept in flight cages (3 × 1 × 1 m) under glasshouse conditions (22 ± 1°C, 60% RH, 16 h : 8 h, light : dark). Bumblebees were fed flowering fast‐cycling *B. rapa* plants, and supplementary nectar (BioGluc, Biobest, Westerlo, Belgium) and pollen (Blütenpollen Multiflora, KoRo Handels GmbH, Berlin, Germany). To avoid contamination during our generations and experiments, plants were removed from the bumblebee cages 2 d before the bioassays. Supplementary pollen and nectar were removed 16 h before bioassays.

### Experimental evolution

**Fig. 1:**
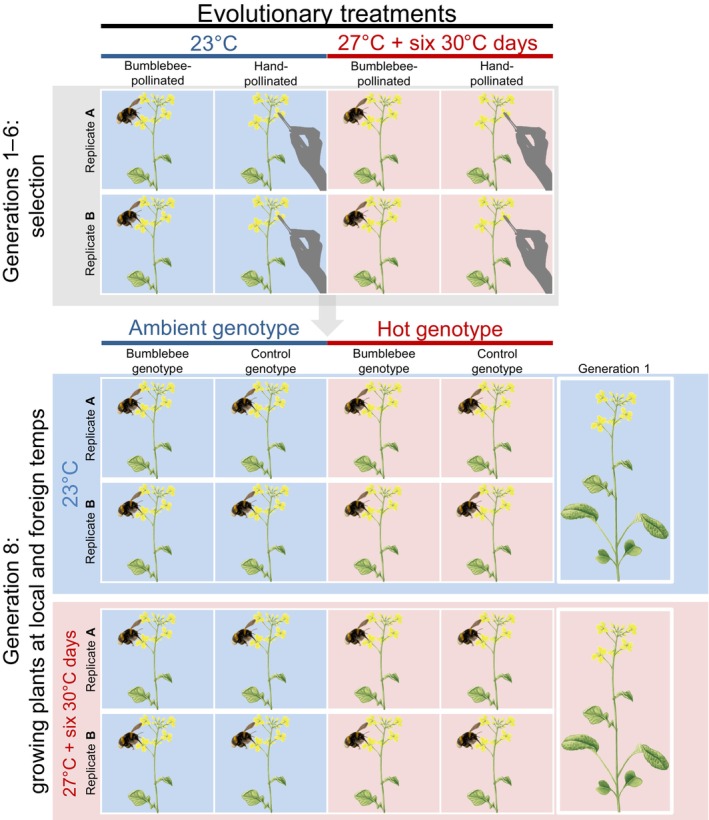
Experimental evolution design: from generations 1–6, plants originating from 98 full‐sibling families (families 1–49 in replicate A, and 50–98 in replicate B) were kept at either 23°C (blue) or at 27°C plus 6 ‘hot’ days of 30°C (red) and were visited by *Bombus terrestris* bumblebees or hand pollinated (control, gray hand). All four treatments started out with the same 98 plant families. In generation 7 (not shown in the figure), all plants were grown at 23°C and hand pollinated to reduce maternal and transgenerational effects from the selection treatments. During the last generation (generation 8), the genotypes ensuing from experimental evolution (pollination and temperature genotypes) and generation 1 plants were grown at both their local and foreign temperatures (23°C, blue; 27°C plus 6 ‘hot’ days of 30°C, red). Evolved plants from all temperature (ambient and hot) and pollination (bumblebees and control) treatments were exposed to visitation by *B. terrestris* bumblebees in the ‘local adaptation assays’.

We performed experimental evolution for six generations (Fig. 1). This experiment was conducted in two glasshouse cabins, one with a constant temperature of 23°C (ambient condition) and one with a constant temperature of 27°C, plus 1 d wk^−1^ at 30°C (during the whole day), repeated six times (hot condition). These temperature parameters are an approximation of the current and mid‐century mean early summer temperatures in northern Switzerland predicted by a model consisting of 68 simulations executed by seven regional climate models driven by nine global climate models (Fischer *et al*., 2022; MétéoSuisse, 2024). Pollinators were kept at ambient conditions, because we assumed that in nature, insects are more flexible in choosing places with optimal temperatures for roosting and nesting. At both ambient and hot conditions, plants were visited by either *B. terrestris* pollinators (*Bombus*‐treated plants) or were hand‐pollinated (control plants, see in the subsequent section). Hand‐pollinated control plants differed not only in mode of pollination from bumblebee‐pollinated plants, but also in the way seeds were carried forward into the next generation (see section below). This was done to use the control plants as representatives of generation one, yet accounting for ‘background effects’ during the evolution experiment (e.g. seed mortality and selection during germination, see in the subsequent section). Therefore, hand‐pollinated control plants were not considered suitable to assess the effects of hand pollination against bumblebee pollination, but merely to assess the evolutionary change caused by bumblebees compared to the starting population.

Altogether, we applied four treatments, where temperature and pollination treatments were combined in a full factorial manner (Fig. 1). We used 98 full‐sib families to constitute the base plant populations for each treatment of our experimental evolution. These 98 families were divided into two replicates (A and B) containing 49 families each; replicates with plants from the same families were used as the starting population of every treatment. Although within one treatment, replicates A and B were submitted to the same conditions, they were never crossed and were allowed to evolve as independent lines.

Every generation, plants were sown in multi‐pot trays filled with a sowing mix consisting of equal parts sterilized sieved topsoil, compost (3 yr‐old), pumice (1–5 mm) and peat (from the Botanical Garden), and allowed to germinate for 8 d in a phytotron, with 24 h light, 22 ± 1°C and 60% RH. On the eighth day, seedlings were re‐potted individually in 7 × 7 × 8 cm pots filled with standardized soil (Patzer Einheitserde, Sinntal, Germany) and taken to either the ambient or hot glasshouse cabin depending on their treatment. Plants in both cabins were watered two times per day, in the morning and evening. Plus, to avoid differences in drought between temperature cabins, plants were checked at least two more times every day, and supplementally watered where needed, to ensure similar levels of soil moisture in plants across cabins. This was done to avoid drought effects, which would have made the interpretation of the results more difficult, although it may have compromised ecological realism (see also the Discussion section). At 24–27 d after sowing, plants were exposed to bumblebees and control plants were hand‐pollinated. Insect visitation happened in the ambient cabin for ambient‐evolving plants and in the hot cabin for hot‐evolving plants, so that temperature conditions also could influence the preference and behavior of the bumblebees.

### Pollination by bumblebees

Each generation, four replicates of 49 plants (two replicates evolving in the ambient cabin and two replicates grown in the hot cabin) belonging to the *Bombus*‐treated group were subjected to pollination by bumblebees (Fig. 1). Within each replicate, plants were randomly arranged in a 7 × 7 matrix inside a flight cage (2.5 × 1.8 × 1.2 m). In this cage, we released 12 individual worker bumblebees sequentially. Each bee was allowed to visit four plants and was then recaptured before releasing the next bumblebee. The limitation to four visits was applied to avoid too many plants being visited by bees and thus a lack of pollen limitation in the plant population. For seed maturation, we kept only plants that had been visited (because the non‐visited plants do not produce any seeds). To do so, we recorded which plants were visited and since the first visited plant did not receive any pollen from another individual, we kept the second, third and fourth visited plants for fruit maturation and seed collection. As a result, we kept 20–27 plants per replicate each generation. All non‐visited plants were discarded.

### Control plants

Every generation, all plants from each replicate belonging to the control groups (two replicates in the ambient cabin and two replicates in the hot cabin) were hand pollinated (Fig. 1). To ensure successful seed production and avoid genetic incompatibilities, we used a pollen mixture for hand pollination. Within each replicate, we collected pollen with a makeup brush from three to five flowers of every plant. Then, this brush was used to pollinate six flowers per plant. This was a control for our evolution environments, which allowed us to check whether each temperature environment (our glasshouse cabins) could cause phenotypic divergence through differential plant mortality, fertility, and germination, or additional transgenerational effects.

### Seed maturation and contribution to the next generation

One month after bumblebee‐ or hand pollination, plants were moved to a different glasshouse, where watering was stopped and plants were dried out so the seeds could mature and be collected. For bumblebee‐pollinated plants, seeds were counted after collection by emptying all seeds from one plant on a white plate, photographing it and using the software ImageJ to count the seed numbers from the pictures. Each generation, we grew 49 plants per replicate from seeds of the previous generation. The contribution of each plant to the next generation was directly proportional to the amount of seeds they produced, and was calculated as follows: 49/(replicate sum of seeds/individual seed number).

For the control plants, each plant contributed one seed to the next generation, but if a plant did not produce seeds due to mortality or unsuccessful reproduction, we replaced that genotype with another plant from a random mother plant that successfully reproduced. Thus, because of the hand pollination and the way seeds were carried into the next generation, our control plants cannot be considered a control for pollinator‐mediated evolution, but a control for glasshouse adaptation or other changes from the starting generation. Indeed, control plants closely resembled plants of generation one in all but one trait (see Supporting Information Notes S1; Table S1).

Plants underwent six generations of selection in the manner described above. To reduce maternal effects, for the seventh generation, we grew plants from all four treatments at ambient temperature and hand‐pollinated them following the same protocol described above for control plants. This time 10 flowers per plant were pollinated instead of 6, so we would have enough seeds for all subsequent tests. The ensuing seeds were collected and a random set was used to grow the eighth generation, in which we assessed trait evolution and conducted bioassays.

### Analysis of evolutionary trait divergence/bioassays

To see if plants diverged in their traits in response to temperature and bumblebee pollination, we randomly selected 36 mother plants from each treatment and replicate of the seventh generation and used their seeds to grow the eighth generation. From each selected mother plant, we grew two half‐sibling plants, one in a glasshouse cabin with a temperature of 23°C (ambient temperature) and one in a glasshouse cabin with a temperature of 27°C plus six weekly 30°C days (hot temperature). In this way, plants from all treatments were reciprocally grown both at the temperature they evolved in and at the opposite temperature, allowing us to test for evolutionary changes in heat‐mediated plasticity and temperature local adaptation. Alongside generation 8, we grew 72 plants from the first generation (G1), 36 in the ambient and 36 in the hot cabin. The protocol for sowing, potting and plant cultivation was the same as described above for every generation. To be able to manage and test all plants (648 plants in total), plantings were divided into six cohorts, planted weekly. Every week we sowed 108 plants, consisting of six plants from each replicate (and six G1 plants). Thus, the effects of planting time were distributed evenly across all treatments.

### Plant phenotyping of flowering, morphology, rewards, and color

From the second week after sowing, we monitored plants every day for the start of flowering. During the fourth week (21–25 d after sowing), we measured plant height (in cm, with a 5 mm precision), number of open flowers and number of leaves. We collected three flowers from every plant, extracted their nectar, and glued their separated petals on an A4 white paper sheet using transparent scotch tape. These paper sheets were then scanned at 600 DPI with a printer (Canon imageRunner Advance DX) and the resulting .JPEG images were analyzed using the software ImageJ. With it, we measured petal area (in cm^2^). The sheets were also photographed under UV light (1 W‐UV Torch, λ = 365 nm and intensity = 6.6 mW cm^−2^) using a photo camera with a UV transmission filter (Nikon D200 digital SLR, with quartz lens: Nikon UV Nikkor f4.5/105 mm with Baader 2″ U‐Filter (310–390 nm UV transmission)), and the area of UV absorbance per petal (in cm^2^) was measured with ImageJ. The area of UV absorbance per petal was divided by the petal area to calculate the relative area of UV absorbance per petal. We extracted nectar by pressing a small 1 μl glass capillary against the nectaries. Since the same capillary was used for all three flowers, the measured amount was then divided by three to calculate nectar per flower. We estimated nectar per plant by multiplying nectar per flower by the number of open flowers.

We collected one extra flower for color measurements. For two petals per collected flower, we measured color reflectance 0.5 mm below the top edge of the petal using a spectrometer (Avaspec‐2048; Avantes, Apeldoorn, the Netherlands) with a fiber optic reflective probe and a Xenon pulsed light source (AvaLight‐XE; Avantes). We followed the same protocol detailed in Traine *et al*. (2024).

### Flower scent collection and quantification

Floral volatiles were collected and quantified via headspace sorption and by using a push‐pull system (Schiestl *et al*., 2018; Ramos & Schiestl, 2019). Scent was collected 21–24 d after sowing in the ambient temperature glasshouse cabin, before all other measurements were done, because damage during plant handling could change the scent emission. The main inflorescence of each plant was encased in a glass cylinder with a silanized surface (sigmacote; Sigma‐Aldrich, Buchs, Switzerland) on the inside. The glasses were sealed at their base with two Teflon plates, which only let the inflorescence stem through a hole (0.5 cm ⌀). Air was pushed through a side opening in the cylinder (fitted with an activated charcoal filter to purify the incoming air) and pulled through another opening containing a glass tube filled with an absorbent (30 mg of Tenax). Both pushing and pulling were performed by pumps at a rate of 120 ml min^−1^. At the start of each collection, we counted the number of flowers encased within the glass cylinders. Collections lasted for 2 h between 11:00–13:00 and 14:00–16:00 h. One sample from an empty glass cylinder was collected as an air control during each collection round.

We quantified flower scent using chromatography with mass selective detection (GC‐MSD). We injected samples into a GC (Agilent 6890N; Agilent Technologies, Santa Clara, CA, USA) by a MultiPurpose Sampler (MPS; Gerstel, Mülheim, Germany) equipped with a Gerstel thermal desorption unit (TDU; Gerstel) and a cold injection system (CIS; Gerstel). The GC was fitted with a HP‐5 column (15 m length, 0.25 mm ID, 0.25 μm film thickness) and helium was used as a carrier gas at a flow rate of 2 ml min^−1^. Scent compounds were desorbed from the Tenax by heating the TDU from 30°C to 240°C at 60°C min^−1^, and holding the final temperature for 5 min. Compounds were then trapped in the CIS at −150°C. For injection, the CIS was heated to 250°C (12° per s) and held at that temperature for 3 min. We identified and quantified each compound with a mass selective detector (Agilent MSD 5975). The mass spectra of each compound were compared with spectra belonging to the library of the National Institute of Standards and Technology (NIST23), and with the spectra of previously analyzed synthetic standards. Quantification (total ion counts) of each compound was performed using a calibration curve for target ions specific to the individual compounds. All scent compounds were standardized in units of picogram per flower per liter sampled air per hour.

### Bumblebee bioassays

We performed bioassays after phenotyping to measure the attractiveness of plants to pollinators and fitness (seed set) of plants grown at their local temperature (temperature they evolved in) vs plants grown at their foreign temperature (temperature they did not evolve in). We tested this separately for bumblebee‐evolved and control plants, using bumblebees for all plants as visitors. Using bumblebees as visitors in both groups allowed us to test whether pollinator‐mediated adaptation led to more seed set in the bumblebee‐evolved (i.e. bumblebee‐genotype) plants compared to control plants with hand pollination (at both temperature regimes).

Within each treatment (*Bombus*‐treated and control), we performed six bioassays testing the attractiveness and fitness (seed number) of ambient‐grown ambient‐evolved (local) vs ambient‐grown hot‐evolved (foreign) plants in ambient conditions, and six bioassays testing the attractiveness and fitness of hot‐grown ambient‐evolved (foreign) vs hot‐grown hot‐evolved (local) plants in hot conditions. It amounted to 24 bioassays in total, spread over 6 wk (four per week). For each bioassay, 24 plants were set up in a 5 × 5 matrix inside a flight cage. Each plant matrix contained 12 ambient‐evolved plants (6 from replicate A and 6 from replicate B) and 12 hot‐evolved plants distributed following an adjusted Latin square layout where repetition of treatments in each row and column was minimized. In each cage, we released six individual worker bumblebees sequentially. Bees were starved for 16 h before the tests and did not have contact with any plants for 2 d before the assays. Each bee was allowed to visit five plants and then recaptured before releasing the next one. For each bumblebee, we recorded which plants were visited, the number of flower visits per plant and the time spent per plant. Since the first visited plant could not have received any pollen, we kept the second, third, fourth and fifth visited plants for fruit maturation and seed collection and discarded the rest. As a result, roughly 50–70% of plants per bioassay were kept for seed collection.

One month after pollination, plants were moved to another cabin where watering was stopped, for the plants to dry and the fruits to mature. Once all the fruits were mature, *c*. 2 wk after the move, they were collected. We counted seeds per plant by opening the fruits and placing all the seeds of each plant on a white plate, photographing them, and using the pictures to count the number of seeds on ImageJ.

### Statistical analyses

We carried out statistical analyses in R (v.4.3.2 × 64, 2023; The R Foundation for Statistical Computing Platform, Vienna, Austria) and Spss v.14 (IBM Corp., Armonk, NY, USA). Generally, we did not adjust *P*‐values for multiple comparisons (Bonferroni correction), because we compared individual traits between treatment groups, and our hypothesis testing was not based on groups of traits (i.e. table‐wide statistical differences). For individual traits, the likelihood of type I errors is independent of the number of comparisons done, and thus, *P*‐value adjustments are not justified. We used generalized linear mixed models (GLMMs) for most analyses. In the models, continuous data were modeled using a Gaussian distribution if normality assumptions were met, a Gamma distribution with a log link function if not, and a Tweedie distribution if data were zero‐inflated. Count data were modeled using a Poisson distribution, a negative binomial distribution with a log link function if data were overdispersed, or a Tweedie distribution if data were zero‐inflated. For proportional data, we used a beta distribution. For the time until flowering, we used a Cox proportional hazards distribution. The fit of all distributions was checked using the function *SimulateResiduals* from the package darhma (Hartig, 2022), except for Cox models, where we tested the proportional hazards assumption using the function *cox.zph* from the package survival (Therneau & Lumley, 2015). All flower scent compounds were log_e_ + 1 transformed before analysis to approach normal distribution, and the compounds limonene, β‐pinene and nonanal were excluded since their amounts were as high in the air controls as in the flower samples. All other trait data were not transformed before analysis.

To discriminate the effects of natural selection from drift in the phenotypic data, in the models analyzing evolutionary trait differences, we assessed whether trait differences were consistent among replicates of a given treatment group by including replicate as a random factor in all models. In the GLMM analysis, a significant effect of one or both of the two factors (pollination genotype and temperature genotype) indicated trait difference between different treatment groups across the replicates, which was seen as an evolutionary change caused by selection.

#### Adaptation/bioassays

Using generalized linear mixed models, we tested the effect of pollination genotype (bumblebee and control), temperature genotype (ambient‐evolved and hot‐evolved), temperature environment (ambient‐grown and hot‐grown) and their interactions on seed number, fruit number and seeds per fruit. In all analyses, we treated the control group as a proxy of generation one, taking into account unwanted glasshouse effects (see the Materials and Methods section). Since in the initial analysis with all factors, pollination genotype was highly significant for seed number (Table S2), we analyzed the bumblebee‐evolved and control plants separately, with temperature genotype, temperature environment and their interaction as factors. To study insect preference and understand the insect behaviors potentially contributing to differences in plant fitness, we ran (generalized) linear mixed models testing the effect of temperature genotype and temperature environment and their interaction (T × E) on pollinator first choice, number of flower visits per plant, time spent per plant and time spent per flower. Cohort and replicate were included as random factors in all analyses. Further, to better understand bumblebee preference within our bioassays, we performed multiple regressions using all tested plants, with bumblebee visits per plant as the dependent variable and plant standardized traits (scaled and centered) used as covariates. To avoid overfitting, models on phenological/visual traits and floral scent compounds were performed separately. To control for multicollinearity, we calculated the Variance Inflation Factor (VIF) of the covariates for every model using the *check_collinearity* function from the package performance and excluded traits with a VIF above 5 in a stepwise manner (Fox & Weisberg, 2019; Palacio *et al*., 2019; Ludecke *et al*., 2021).

#### Evolutionary trait divergence

We analyzed the differences in individual traits (plant height, days to flower, flower number, leaf number, petal area, nectar volume, UV relative area (ratio of UV‐absorbing area), UVA/B and yellow relative diffuse reflectance (RDR, a relative percentage of reflectance), each individual flower scent compound, the sum of all flower scent compounds) using (generalized) linear mixed models. Each trait was tested as a dependent variable, with pollination genotype, temperature genotype, temperature environment, and their interaction as fixed factors. These analyses were also done for bumblebee‐evolved and control plants alone, to better detect evolutionary divergence in response to temperature. Each trait was tested against temperature genotype, temperature environment, and their interaction. Planting week (cohort) and replicate were included as random factors. We also performed (generalized) linear mixed models testing for differences in individual traits between our two control treatments (hot and ambient) and generation 1 plants, including cohort as a random factor.

#### Evolutionary rates

To assess evolutionary rates, we used the Haldane index for synchronic comparisons for all traits between plants from the last and first generation (Hendry & Kinnison, 1999). To obtain this, we first calculated the absolute difference between the mean at G8 and the mean at G1 per replicate for all traits of ambient‐grown plants. We used absolute values because not all trait averages increased with generations, but we wanted to test for absolute change independent of its direction. We then calculated the standard pooled deviation per replicate for all traits; this was achieved by using the following formula:

$$\text{SDpooled}=\sqrt{\frac{\left(\mathrm{SD}1^{2}+\mathrm{SD}2^{2}\right)}{2}}$$

where SD1 is the standard deviation for a trait from a replicate of G8 and SD2 is the standard deviation for a trait from G1. Lastly, we calculated the Haldanes per replicate for every trait using: Haldanes = ((absolute mean difference (G8 vs G1))/SDpooled)/6. Using Haldanes per replicate for every trait as a response variable, we ran linear mixed models to test the effect of temperature genotype, visitation, trait category (visual or scent) and their interaction on evolutionary rates. Replicate and trait were added as random factors.

#### The evolution of heat‐mediated phenotypic plasticity

Using plants from all evolved treatments grown at both temperatures, we used (generalized) linear mixed models to test the effect of pollination genotype, temperature genotype and temperature environment and their interactions using each trait as our dependent variable (as stated above). In these analyses, a significant temperature environment effect indicates plasticity for that trait, while significant interactions would indicate that the plastic responses differed in magnitude or direction between treatments. To further detect differences in plasticity between evolved treatments, we obtained the reaction norms in response to temperature for each trait by calculating the difference between the trait values of ambient‐grown and hot‐grown half‐sibling plants (Ramos & Schiestl, 2020; Wang *et al*., 2022). Using the reaction norms of each trait as response variables, we used linear mixed models to test the effect of temperature genotype, pollination genotype and their interaction. Cohort and replicate were included as random factors. These models were performed for the whole dataset, and also within bumblebee‐evolved and control plants alone. There, we tested the effect of temperature genotype on trait reaction norms.

#### The evolution of honest signaling

Honest signaling in flowers is defined as an association between the amount of reward and the strength of a particular flower signal (Knauer *et al*., 2021). We analyzed the differences in honest signals between treatments using (generalized) linear mixed models. In the models, nectar per flower was tested as a dependent variable, with each individual plant trait as a covariate (plant height, flower number, leaf number, petal area, UV relative area (ratio of UV‐absorbing area), UVA RDR (relative diffuse reflectance and a relative percentage of reflectance), UVB RDR, yellow RDR, each individual flower scent compound, the sum of all flower scent compounds and the sum of all aromatic flower scent compounds), and with pollination genotype, temperature genotype and temperature environment as fixed factors. Individual models were performed for each trait, and included the interactions between each tested trait, pollination genotype, temperature genotype and temperature environment. A significantly positive correlation between nectar production and a trait would indicate this trait was an honest signal, while a significant interactive effect may indicate that honest signaling varied between temperature environments, temperature genotypes and/or pollination genotypes. Planting week (cohort) and replicate were included as random factors. These analyses were also done for bumblebee‐genotype plants and control plants alone, where nectar per flower was tested against each trait, temperature genotype, temperature environment and their interaction. For flower size and phenylacetaldehyde emission, two plant traits that have been shown in the past to be honest signals in this system (Knauer *et al*., 2021), we also performed Pearson correlations for honest signaling in ambient‐genotype and hot‐genotype plants within each pollination genotype and each temperature environment.

## Results

### Adaptation during the experiment (question 1)

**Fig. 2:**
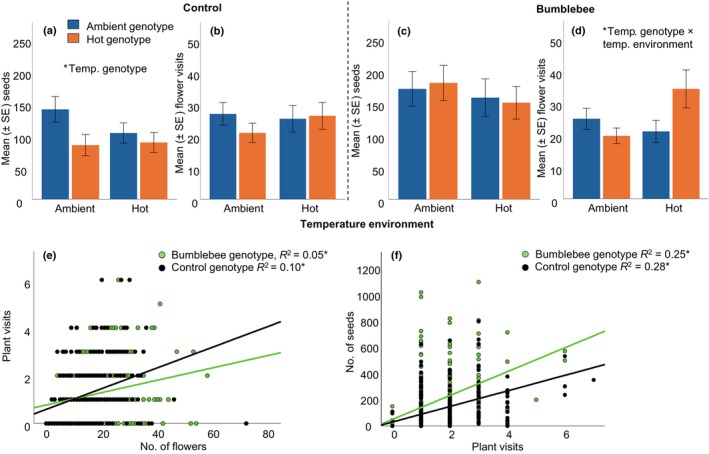
Plant adaptation to bumblebees reduced the negative effects of temperature on seed set. Meanwhile, in control‐genotype plants, hot‐genotype plants had reduced seed sets (significant effect of temperature genotype), but this was not the case in bumblebee‐genotype plants. When analyzed together, bumblebee‐genotype plants had higher seed set compared to control plants, irrespective of temperature environment or temperature genotype (Supporting Information Table S1). Bar plots show the mean (±SE) seeds per plant and flower visits of bumblebees on control plants (a, b) and on bumblebee‐genotype plants (c, d), for both ambient‐ (blue bars) and hot genotypes (orange bars), in the ambient and hot environment (for statistical values see Tables S1, S2). Scatter plots show the association between the number of flowers and visitation by bumblebees (e) and visits of bumblebees and the number of seeds produced by the plants (f) for both the bumblebee (green trend line) and control group (black trend line). For bar plots, error bars show SE of the mean; asterisks indicate significant (*P* < 0.05) effects of generalized linear mixed models (a–d) and linear regressions (e, f; for stats values see Table S2); sample sizes were 64–72 per treatment.

We analyzed local adaptation by counting seed set after bumblebee pollination of the plants grown in the temperature environment they have evolved in (local) as well as the environment they did not evolve in (foreign); we also assessed adaptation to bumblebees, by counting seed set of plants that had evolved with bumblebees compared to control plants. We found no indication of local adaptation to the temperature environment (i.e. significant interaction between temperature genotype × temperature environment) for either bumblebee or control‐genotype (Tables S2, S3). We did find adaptation to bumblebees, indicated by a significantly (i.e. 63%) higher seed production in bumblebee‐genotype plants compared to control‐genotype plants (Fig. 2; Tables S3, S4; in all analyses, control plants were used as a proxy of generation one). For the effects of temperature on seed set, the bumblebee and control treatments differed. Bumblebee‐evolved plants showed no negative effects of temperature on seed number, fruit number or seeds per fruit (there was no significant effect of either temperature genotype, temperature environment or their interaction, G × E; Fig. 2; Table S2). Regarding bumblebee visitation, there was also no effect of any factor on the number of plants visited by bumblebees; however, bees visited more flowers from hot bumblebee‐genotype plants in the hot environment (Fig. 2d; Table S3). For the control plants, however, temperature genotype had a significant effect on seed number; hot‐evolved control plants produced significantly fewer seeds than ambient‐evolved control plants when they were grown and tested at ambient temperature, but they produced equally few seeds when grown in the hot environment (Fig. 2a; Table S4). Regarding bumblebee foraging, there were no effects of temperature, pollination genotype, temperature environment or their interaction on bumblebee preference in the control treatment (Fig. 2b; Table S3).

### Traits determining plant attractiveness

Using our data of plant visitation and traits, we analyzed which traits most strongly explained plant attractiveness to bumblebees. Whereas none of the scent compounds or color signals showed a significant association with bee visits, four morphological traits were positively associated with plant attractiveness: plant height, number of flowers, number of leaves, and petal area (Fig. 2e; Table S5). Of these four traits, the number of flowers showed the strongest association with bee visitation (Fig. 2e) and was thus assumed to be the most important factor in plant attractiveness to bees. The number of visits was also strongly associated with the number of seeds (Fig. 2f).

### Trait evolution (question 2)

**Fig. 3:**
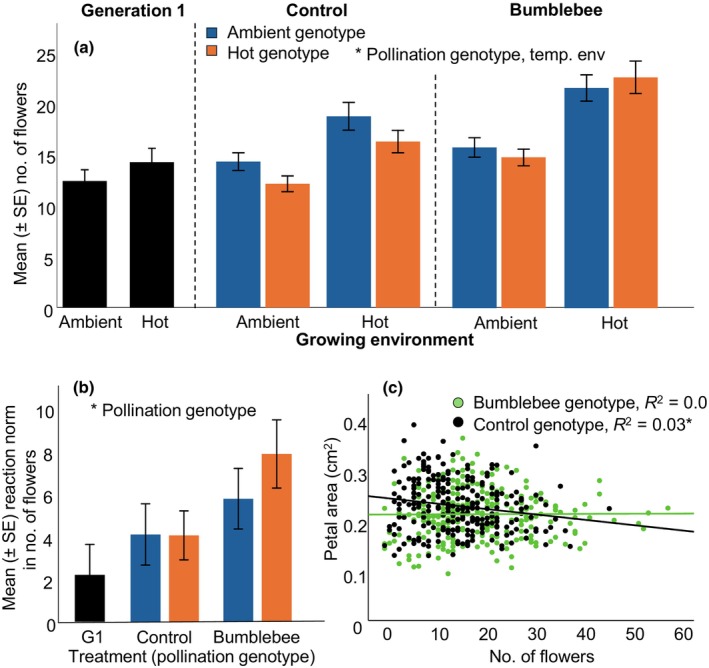
Evolutionary increase in number of flowers (a); (a) shows that bumblebee‐genotype plants, and plants in the hot environment produced more flowers (significant effects of pollination genotype and temperature environment). Evolutionary increase in heat‐mediated plasticity in the number of flowers in bumblebee‐genotype plants (b), evidenced by the increase in the reaction norm (difference in number of flowers between environments). The association between the number of flowers and flower size in control‐ and bumblebee‐genotype plants (c) shows that these traits were negatively associated only in the control group plants. For bar plots, error bars show SE of the mean; asterisks indicate significant (*P* < 0.05) effects of the factors in the generalized linear mixed models (for values see Supporting Information Table S7); sample sizes were 30–72 per treatment. (c) Data were analyzed by linear regression; bumblebee genotype: β = 0.01, *P* = 0.924, control genotype: β = −0.17, *P* = 0.006; *n* = 252 per treatment.

We analyzed trait evolution by comparing trait values in bumblebee‐ and temperature‐evolved plants and control plants. For the number of flowers, we found that bumblebee‐evolved plants had significantly more flowers than control plants (highly significant effect of pollination genotype in our combined analysis, including all factors (pollination genotype, temperature genotype and temperature environment) and their interactions; Fig. 3a; Tables S6, S7). To ease the interpretation of the data, we analyzed the two pollination treatments separately as well. This analysis showed that in the control treatment, the hot‐genotype plants had fewer flowers, suggesting that hot temperature had inhibited the evolution of more flowers in this treatment group (Fig. 3, *X* ^2^ = 5.03, *P* = 0.025). No such effect was found in the bumblebee‐evolved plants (Table S7).

**Fig. 4:**
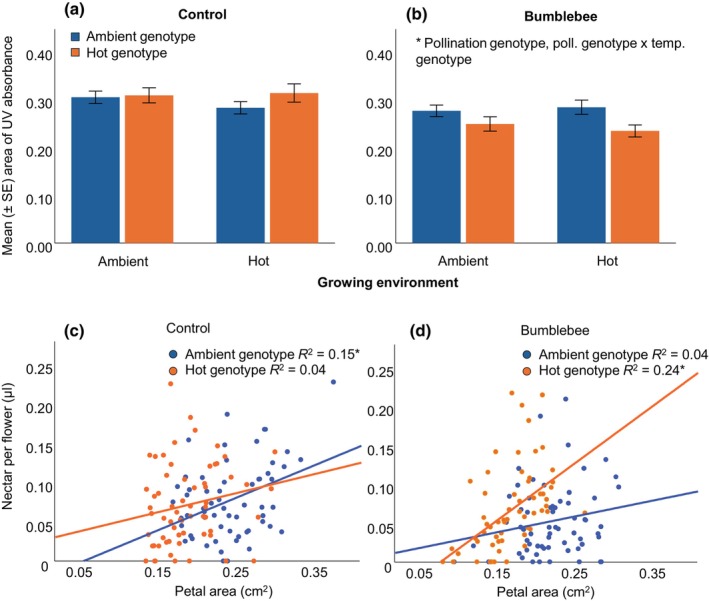
The evolutionary patterns and plasticity in UV absorbance in control‐ (a) and bumblebee‐genotype plants (b) and the association between flower size and nectar, for plants in their ‘native’ environments in control‐ (c) and bumblebee‐genotype plants (d). The graphs show that in bumblebee‐genotyped plants, flower size becomes a stronger honest signal in hot‐genotype plants, and UV absorbance decreases in hot‐genotype plants. Error bars in bar plots show SE of the mean. Asterisks indicate significant (*P* < 0.05) effects of the factors in the generalized linear mixed models (for value see Supporting Information Table S7). (c) Ambient: β = 0.38, *P* = 0.002, hot: β = 0.19, *P* = 0.137; (d) ambient: β = 0.19, *P* = 0.135, hot: β = 0.49, *P* < 0.001; data in (c, d) were analyzed using linear regression, *n* = 59–62.

For the other traits, in the bumblebee‐evolved plants, the petal area and nectar per flower decreased, flowering time was delayed, and several scent compounds decreased, possibly as a trade‐off with the number of flowers. UV absorbance area decreased while UV reflectance increased, especially in bumblebee‐hot‐evolved plants (Fig. 4a,b); the number of leaves increased in bumblebee‐hot‐evolved plants only (Table S7).

### Heat‐mediated phenotypic plasticity (question 3)

Most traits showed a significant effect on the growing environment, evidencing strong plasticity triggered by elevated temperature (Table S7). Heat‐mediated plasticity increased plant height, leaf number, flower number and nectar per plant, whereas it sped up flowering and decreased petal area and most floral scent compounds (Table S7). For 15 traits, plasticity was in the same direction as trait evolution (i.e. the effect of pollination genotype or temperature genotype, see above, Table S7), whereas for four traits, it was the opposite (Table S7). Comparing the reaction norms showed that plasticity (i.e. increase) in the number of flowers increased significantly in the bumblebee‐genotype plants, whereas plasticity (i.e. decrease) in petal area significantly decreased in bumblebee‐genotype plants (Fig. 3b; Tables S8, S9). Plasticity in flowering time evolved differently between temperature‐ and pollination genotypes, as it did not change in bumblebee‐hot genotypes, whereas it decreased in control hot genotypes (Tables S8, S9, *X* ^2^ = 4.43, *P* = 0.035). Plasticity (i.e. increase) in nectar per plant evolved differently between temperature‐ and pollination genotypes. It increased significantly in bumblebee‐hot‐genotype plants (Tables S8, S9). For floral scent, we showed a decrease in plasticity in 1‐butene‐4‐isothiocyanate in bumblebee‐genotype plants, and a decrease in plasticity for 2‐amino benzaldehyde, methyl anthranilate, benzyl nitrile, methyl salicylate, (*E,E*)‐α‐farnesene, and the total volatile emission in bumblebee‐ambient‐genotypes (Tables S8, S9).

### Reward and signals (question 4)

Whereas flower size (petal area) became smaller in bumblebee‐evolved plants, the association between flower size and number of flowers differed between bumblebee‐ and control plants. We found a negative association between number and size of flowers, but only in the control group, whereas in the bumblebee‐evolved plants, this association was not significant anymore (Fig. 3c).

Overall, a significant association between nectar per flower and floral signals was found for flower size and several aromatic scent compounds, including phenylacetaldehyde, a compound previously identified as an honest signal (Knauer *et al*., 2021; Table S10). The association between signal and reward varied between pollination‐ and temperature genotypes and temperature environments (Table S10). For flower size, the association with nectar in the bumblebee‐evolved plants was only significant in the hot genotypes, in both environments (Figs 4d, S1C,D). In the control group, on the contrary, the association was significant in both ambient‐ and hot genotypes, but only in the ambient environment (Fig. S1A). For phenylacetaldehyde (PAA), the only significant association with nectar was found in the bumblebee‐evolved plants, in the ambient genotype and ambient environment (Fig. S1G). This suggests that whereas PAA is an honest signal in bumblebee‐evolved plants in ambient genotypes only, flower size becomes the primary honest signal when evolving in hot environments (Fig. 4c,d), that is, signaling in hot environments evolves to become more visual, which is also caused by the strong reduction of many aromatic compounds in the hot environment.

### Evolutionary rates

Evolutionary rates were significantly higher for bumblebee‐evolved plants, but did not differ according to trait categories, temperature treatment or their interactions (Table S11).

## Discussion

Biotic pollination is a key factor for seed set in the majority of angiosperms (Ollerton *et al*., 2011); however, the effects of global warming on plant–pollinator interactions have primarily been investigated in the context of phenological shifts and potential desynchronization between plants and their pollinators (Memmott *et al*., 2007; Freimuth *et al*., 2022). Nevertheless, temperature stress also impacts biotic interactions through changing flower traits and fertility in plants, as well as the behavior of pollinators, often leading to reduced fitness (Descamps *et al*., 2021a; Cordeiro & Dötterl, 2023a; de Manincor *et al*., 2023; Traine *et al*., 2024). Here, we confirm that temperature stress leads to reduced seed set in an outcrossing plant with bumblebee pollination, but also show that the negative effects can be compensated by rapid adaptive evolution under selection by bumblebee pollinators. This adaptive evolution was primarily enabled by an increased number of open flowers on pollination day and an augmented heat‐induced plastic increase in the number of flowers. Our results show that adaptive evolution enabled by biotic pollination can mitigate the negative effects of environmental stress in a glasshouse experiment, which avoided additional stressors such as drought. Whether such adaptive evolution will also ensue in nature depends on the impact of other factors such as limited water availability potentially associated with heat, as well as trade‐offs caused by more open flowers, such as the increased attraction of herbivores.

The fact that a warming climate can cause declines in fitness in many organisms by disrupting local adaptation and/or causing temperature stress is well established (Alexander *et al*., 2015; Anderson & Wadgymar, 2020; Descamps *et al*., 2021a; Hamann *et al*., 2021b; de Manincor *et al*., 2023). A major question is, however, whether evolutionary rescue is possible, and how fast plants can evolve new traits to be better adapted to the altered climate (Csillery *et al*., 2020; Gérard *et al*., 2020; Hamann *et al*., 2021a; Martin *et al*., 2023). Few studies have addressed this question so far (Rineau *et al*., 2019), and some of them found rapid adaptation to climate‐change‐related environmental factors such as drought or temperature (Frenck *et al*., 2013; Thompson *et al*., 2013; Franks *et al*., 2014, 2018; DeMarche *et al*., 2018; Sun *et al*., 2020; Johnson *et al*., 2022). Other studies, however, indicated that adaptive responses are lagging behind climate change (Wilczek *et al*., 2014), depend on population history or the interaction of ecological factors (Frenck *et al*., 2013; Santos *et al*., 2023), or are constrained by genetic correlations (Etterson & Shaw, 2001). In our study, we showed with an evolution experiment that increased temperatures triggered adaptive evolution in plants when they were pollinated by bees, decreasing the negative effects of temperature stress on seed set. Selection mediated by bumblebees led to adaptive evolution in both ambient and hot temperatures, and adaptation was not delayed or constrained by temperature.

Our control plants resembling the starting population had lower seed set in the hot genotypes in both environments, and in the ambient genotype in the hot environment, which was evidently not related to bumblebee visitation during the bioassays, as bumblebees displayed no preference for any genotypes in any environment. Hence, the reduced seed set was probably caused by the reduced fertility of these genotypes in hot environments. A reduction in seeds per fruit in hot environments was already shown in our earlier studies focusing on plasticity in *B. rapa* (Traine *et al*., 2024), and an evolved reduction of fertility under elevated temperatures was found earlier in *Brassica napus* (Frenck *et al*., 2013). Our control plants were not under selection via pollination and only under mild selection via differential plant mortality, sterility or germination, which was evidently not strong enough to overcome the negative effects of heat stress.

The most fitness‐relevant evolutionary change in plant traits in our experiment was the increase in the number of flowers open at pollination day in the bumblebee‐genotype plants. An increase in the number of open flowers is also a plastic response to warmer environments in *B. rapa* (Traine *et al*., 2024) and likely related to accelerated stem growth in warmer growth conditions documented in *Arabidopsis thaliana* (Lee *et al*., 2020). The evolutionary increase was most likely driven by bumblebee‐mediated selection on ‘number of flowers’ which is commonly found in insect‐pollinated plants (Caruso *et al*., 2019; Rusman *et al*., 2025). As a consequence, plants in hot environments were equally attractive and produced the same number of seeds as those in ambient environments.

We also found an increase in temperature‐driven plasticity in the number of open flowers, with bumblebee‐genotype plants inducing even more open flowers in the hot environment. Indeed, theory predicts that organisms in new environments should first evolve increased plasticity when plasticity is adaptive (Lande, 2009) and evolution may follow the direction of plasticity in this scenario (Noble *et al*., 2019). The number of flowers was adaptive in our experiment in the bumblebee‐evolutionary group, because more flowers were associated with more visits by the bees (Fig. 2). In our experiment, for most of the traits where we detected significant plasticity as well as evolutionary change, the direction of plasticity was the same as that of evolution, which suggests that plasticity is adaptive for most traits in this system. This finding aligns with the ‘plasticity‐first’ hypothesis, suggesting adaptive evolution follows the direction of plasticity, building on the presence of heritable variation in a trait in the same dimension as plasticity (Garland & Kelly, 2006; Noble *et al*., 2019; Ramos & Schiestl, 2020).

Despite the mean flower size decreasing in bumblebee‐evolved plants, the association between size and number became less negative. This indicates that although plants produced more flowers, they were bigger than expected from the trade‐off between ‘size and number’, indicating a trend toward more and proportionally larger flowers in bumblebee‐genotype plants. Besides the size of flowers, we also reported changes in other floral signals such as the UV absorption, which decreased (i.e. UV reflection increased) in the bumblebee‐evolved plants, and even more so in bumblebee‐hot‐evolved plants. UV absorption is caused by UV‐absorbing pigments (i.e. flavonols), commonly found in the center of the flower, forming the so‐called bullseye (Koski *et al*., 2024). UV‐absorbing pigments have different functions and play a role in heat absorption, protection against UV radiation, desiccation resistance, and pollinator attraction (Luethi *et al*., 2022; Todesco *et al*., 2022). Latitudinal patterns in UV absorption among plant populations have been found and plants in colder environments tend to have larger bullseyes and thus lower UV reflection (Koski & Ashman, 2016; Todesco *et al*., 2022). Similarly to our results, Gervasi & Schiestl (2017) found an increase in UV reflectance in bumblebee‐pollinated plants after nine generations of experimental evolution at ambient temperatures. These findings experimentally confirm that patterns of UV absorption/reflection are shaped by a combination of biotic and abiotic selection, likely because of the multifunctional nature of UV‐absorbing pigments.

For floral scent, we showed that increased temperature reduced the amount of most floral scent compounds through plasticity, and of a few through evolution. Plastically reduced scent in hotter temperatures was shown before (Cordeiro & Dötterl, 2023b; Traine *et al*., 2024; Rusman *et al*., 2025), but compound‐specific increases in floral volatiles in Mediterranean plants kept at hot temperatures were detected by other studies (Farre‐Armengol *et al*., 2014). As a consequence of reduced floral aromatic compound emission and a lowered trade‐off between size and number of flowers, honest signaling (i.e. the association between signals and reward) evolved to become more visual in hot‐temperature genotypes, which is evidenced by a stronger association between flower size and nectar in hot‐ and bumblebee‐genotype plants, and a lack of association between nectar and phenylacetaldehyde in these plants.

Additional environmental stressors, such as drought, can accompany warming temperatures (Trenberth *et al*., 2014). Much like rising temperatures, drought can affect plant metabolism, induce plastic responses, mediate changes in biotic interactions, and ultimately alter plant evolutionary trajectories (Descamps *et al*., 2021b; Rauschkolb *et al*., 2022). Drought was shown to reduce photosynthetic rates (hindering plant resource acquisition) by inducing stomatal closure, which prevents carbon assimilation (Feller, 2016). Drought can further lead to plastic changes in pollinator advertisement traits. Under drought, plants can produce fewer and smaller flowers (Burkle & Runyon, 2016; Kuppler & Kotowska, 2021) that emit a qualitatively different scent blend (Campbell *et al*., 2019; Rering *et al*., 2020). Floral rewards, like nectar and pollen, can also be reduced because of drought (Waser & Price, 2016; Phillips *et al*., 2018). Such changes, in turn, have been shown to reduce pollinator attraction and plant reproductive output (Rering *et al*., 2020). Nonetheless, some plant plastic responses to drought may be adaptive (Lambrecht *et al*., 2017). For instance, smaller flowers may mitigate the physiological costs of drought by reducing transpiration loss (Galen, 1999; Teixido *et al*., 2016). In our experiments, we avoided any covariance between temperature and drought by additional watering in the hot‐treatment plants. This may sacrifice some ecological realism, but whereas temperature increases are likely for most regions of the world in the face of global warming, precipitation and hence drought are more variable, with several regions of the world being predicted to have increased precipitation (IPCC AR6WG I SPM). Therefore, while drought is clearly an important ecological factor driving adaptive evolution (Franks *et al*., 2007), it is not clear how climate change will affect drought in different regions worldwide, justifying our focus on temperature and its interaction with biotic pollination in this study.

In conclusion, our results highlight how biotic pollination, by imposing specific selection on key traits, can impact the way plants adapt to climate change. Although our results clearly show that adaptive evolution can effectively reduce the negative effects of heat on plant fitness, our experiments were conducted in a glasshouse environment and therefore did not reflect the complex interaction of factors typically found in nature, or challenge the plants with potential ecological trade‐offs (O'Hara *et al*., 2016; DeMarche *et al*., 2018). An obvious trade‐off of an increased number of flowers may be the increased attractiveness of such plants to herbivores, potentially combined with reduced investment in antiherbivore defense, which could have prevented or slowed down the adaptive evolution under more ecologically complex field conditions. Although experimental evolution with few factors allows a determination of the causal factors of evolutionary change, it should be paired with studies encompassing ecological realism to improve the predictability of adaptive evolution in response to climate change in nature (Rineau *et al*., 2019; Schiestl, 2024).

## Competing interests

None declared.

## Author contributions

FPS, JT and QR designed the study; JT and QR performed the experiment; JT, QR and FPS analyzed the data and wrote the paper. JT and QR contributed equally to this work.

## Disclaimer

The New Phytologist Foundation remains neutral with regard to jurisdictional claims in maps and in any institutional affiliations.

## Supporting information


**Fig. S1** The effect of pollinator‐mediated evolution and temperature environment on the honest signaling of ambient‐ and hot‐genotype plants.
**Notes S1** Comparison of G1 and control group.
**Table S1** Comparison between the control groups and G1.
**Table S2** The effects of temperature‐ and pollinator‐mediated evolution on plant fitness and pollinator preference, in local and foreign temperature environments.
**Table S3** The interactive effects of temperature environment and temperature evolution on plant fitness and pollinator preference, in bumblebee‐ and control‐genotype plants.
**Table S4** Average fitness and pollinator preference for plants tested at their local vs foreign temperature.
**Table S5** Bumblebee preference for plant traits.
**Table S6** The effects of temperature‐mediated plasticity, temperature‐mediated evolution, and pollinator‐mediated evolution on plant traits.
**Table S7** Plant trait divergence in response to temperature environment, temperature‐mediated evolution, and pollination‐mediated evolution.
**Table S8** The evolution of plant temperature‐mediated reaction norms.
**Table S9** The effects of temperature‐ and pollinator‐mediated evolution on plant temperature reaction norms.
**Table S10** Plastic and evolutionary changes in honest signaling.
**Table S11** The evolutionary rates of plant traits.
Please note: Wiley is not responsible for the content or functionality of any Supporting Information supplied by the authors. Any queries (other than missing material) should be directed to the *New Phytologist* Central Office.

## Data Availability

Data are accessible under Dryad Dataset doi: https://datadryad.org/dataset/doi:10.5061/dryad.2ngf1vj15
