# Supplementary material for: Adaptive evolution can mitigate the negative effects of temperature stress on plant–pollinator interactions
Source: New Phytol. 2025 Nov 8;249(1):554–68. doi: 10.1111/nph.70705 (PMC12676098; doi:10.1111/nph.70705)
Supplement: Supplementary file 1 — Fig. S1 The effect of pollinator‐mediated evolution and temperature environment on the honest signaling of ambient‐ and hot‐genotype plants. Notes S1 Comparison of G1 and control group. Table S1 Comparison between the control groups and G1. Table S2 The effects of temperature‐ and pollinator‐mediated evolution on plant fitness and pollinator preference, in local and foreign temperature environments. Table S3 The interactive effects of temperature environment and temperature evolution on plant fitness and pollinator preference, in bumblebee‐ and control‐genotype plants. Table S4 Average fitness and pollinator preference for plants tested at their local vs foreign temperature. Table S5 Bumblebee preference for plant traits. Table S6 The effects of temperature‐mediated plasticity, temperature‐mediated evolution, and pollinator‐mediated evolution on plant traits. Table S7 Plant trait divergence in response to temperature environment, temperature‐mediated evolution, and pollination‐mediated evolution. Table S8 The evolution of plant temperature‐mediated reaction norms. Table S9 The effects of temperature‐ and pollinator‐mediated evolution on plant temperature reaction norms. Table S10 Plastic and evolutionary changes in honest signaling. Table S11 The evolutionary rates of plant traits. Please note: Wiley is not responsible for the content or functionality of any Supporting Information supplied by the authors. Any queries (other than missing material) should be directed to the New Phytologist Central Office. [file NPH-249-554-s001.docx]

New Phytologist Supporting Information

Article title: **Adaptive evolution can mitigate the negative effects of temperature stress on plant-pollinator interactions**

Juan Traine^1^, Quint Rusman^1^, Florian P. Schiestl

^1^These authors contributed equally

Department of Systematic and Evolutionary Botany, University of Zürich, Switzerland

Article acceptance date: 6 October 2025

**Results S1.**

Comparison of G1 and control group: we investigated whether control plants differed from plants of generation one (G1) to see whether the way control plants were treated (hand-pollination, one seed per plant carried further in the next generation, see methods section) led to significant evolutionary change, and whether control plants could serve as a surrogate of plants of generation one in the analysis, yet controlling for background evolutionary effects. Indeed, almost all traits were not significantly different between plant of generation one and both ambient- and hot-genotype of the control group (Table S1). The only exceptions were flowering time and two scent compounds; ambient-evolved control plants flowered significantly later than G1 plants (average of ca. half a day, Table S1). Thus, unwanted selection by greenhouse conditions was apparently weak and control plants resembled G1 plants closely.

**Table S1.** **Comparison between the control groups and G1.** Trait values (mean ± S.E.) and the output of (generalized) linear mixed models showing that plants of generation one and the control group differ only in three traits, so that the control group resembles plants of generation one to a large degree. Bold values indicate results where *P* ≤ 0.05. Underlined values indicate results where *P* ≤ 0.07. Letters indicate significant differences at α = 0.05 based on Tukey’s post hoc tests between trait values. The control plants were for 6 generation either kept at 23^o^C (ambient) or at 27°C plus 6 “hot” days of 30^o^C (hot), and hand pollinated. Scent emission is expressed as pg / flower / l / h. UV relative area calculated by dividing the UV-absorbing surface of the petal (in cm^2^) by the total petal area (in cm^2^). RDR (relative diffuse reflectance) indicates the relative percentage of reflectance.

|  | Generation 1 | Ambient Ctrl | Hot Ctrl | χ^2^ | DF | P |
| --- | --- | --- | --- | --- | --- | --- |
| Height (cm) (N =171) | 34.56 ± 0.93 | 37 ± 0.67 | 35.89 ± 0.7 | 5.7 | 2 | 0.058 |
|  | a | a | a |  |  |  |
| Leaf number (N =171) | 8.77 ± 0.44 | 9.52 ± 0.3 | 8.68 ± 0.34 | 3.09 | 2 | 0.213 |
|  | a | a | a |  |  |  |
| Flower number (N =171) | 12.35 ± 1.09 | 14.27 ± 0.87 | 12.1 ± 0.77 | 3.99 | 2 | 0.136 |
|  | a | a | a |  |  |  |
| Nectar (µl) (N =156) | 0.07 ± 0.01 | 0.08 ± 0.01 | 0.06 ± 0.01 | 2.42 | 2 | 0.299 |
|  | a | a | a |  |  |  |
| Nectar per plant (µl) (N =144) | 0.95 ± 0.18 | 1.23 ± 0.13 | 0.89 ± 0.09 | 4.49 | 2 | 0.106 |
|  | a | a | a |  |  |  |
| Days to flower (N =178) | **18.72 ± 0.13** | **19.39 ± 0.16** | **19.03 ± 0.13** | **8.46** | **2** | **0.015** |
|  | **a** | **b** | **ab** |  |  |  |
| Petal area (cm^2^) (N =157) | 0.25 ± 0.01 | 0.25 ± 0.01 | 0.26 ± 0 | 0.28 | 2 | 0.870 |
|  | a | a | a |  |  |  |
| UV relative area (ratio) (N =157) | 0.29 ± 0.02 | 0.3 ± 0.01 | 0.31 ± 0.02 | 0.17 | 2 | 0.921 |
|  | a | a | a |  |  |  |
| UVA RDR (%) (N =156) | 70.54 ± 3.40 | 73.04 ± 2.11 | 68.43 ± 1.87 | 3.16 | 2 | 0.206 |
|  | a | a | a |  |  |  |
| UVB RDR (%) (N =156) | 72.03 ± 2.37 | 70.93 ± 1.92 | 71.3 ± 1.71 | 0.07 | 2 | 0.967 |
|  | a | a | a |  |  |  |
| Yellow (%) (N =156) | 81.34 ± 1.50 | 81.96 ± 1.11 | 80.63 ± 1.14 | 0.99 | 2 | 0.608 |
|  | a | a | a |  |  |  |
| Benzaldehyde (N =154) | 3.49 ± 0.56 | 3.15 ± 0.39 | 3.01 ± 0.25 | 0.32 | 2 | 0.852 |
|  | a | a | a |  |  |  |
| 1-Butene-4-isothyocyanate (N =154) | **0.80 ± 0.13** | **0.52 ± 0.07** | **0.99 ± 0.15** | **13.38** | **2** | **0.001** |
|  | **ab** | **b** | **a** |  |  |  |
| Methyl benzoate (N =154) | 0.36 ± 0.06 | 0.55 ± 0.1 | 0.42 ± 0.07 | 5.17 | 2 | 0.075 |
|  | a | a | a |  |  |  |
| Phenylethyl alcohol (N =154) | 0.15 ± 0.09 | 0.07 ± 0.01 | 0.07 ± 0.01 | 4.99 | 2 | 0.082 |
|  | a | a | a |  |  |  |
| 2-Amino benzaldehyde (N =154) | 9.44 ± 2.93 | 9.91 ± 1.53 | 11.3 ± 1.69 | 1.83 | 2 | 0.400 |
|  | a | a | a |  |  |  |
| p-Anisaldehyde (N =154) | 0.07 ± 0.02 | 0.1 ± 0.02 | 0.09 ± 0.02 | 1.03 | 2 | 0.596 |
|  | a | a | a |  |  |  |
| Methyl anthranilate (N =154) | 0.88 ± 0.26 | 1.3 ± 0.22 | 0.93 ± 0.17 | 4.11 | 2 | 0.128 |
|  | a | a | a |  |  |  |
| *(Z)-*3-Hexen-1-ol acetate (N =154) | 0.58 ± 0.10 | 0.54 ± 0.09 | 0.56 ± 0.08 | 0.6 | 2 | 0.742 |
|  | a | a | a |  |  |  |
| Phenylacetaldehyde (N =154) | 0.74 ± 0.52 | 0.22 ± 0.06 | 0.22 ± 0.05 | 2.66 | 2 | 0.264 |
|  | a | a | a |  |  |  |
| Benzyl nitrile (N =154) | 1.21 ± 0.35 | 0.95 ± 0.14 | 1.01 ± 0.17 | 0.05 | 2 | 0.974 |
|  | a | a | a |  |  |  |
| Methyl salicilate (N =154) | **0.29 ± 0.05** | **0.38 ± 0.04** | **0.19 ± 0.02** | **24.21** | **2** | **<0.001** |
|  | **ab** | **a** | **b** |  |  |  |
| Indole (N =154) | 3.19 ± 1.09 | 2.64 ± 0.47 | 2.89 ± 0.56 | 0.96 | 2 | 0.619 |
|  | a | a | a |  |  |  |
| *(E,E)-*α-Farnesene (N =154) | 9.50 ± 1.88 | 8.36 ± 0.75 | 9.37 ± 0.85 | 0.73 | 2 | 0.693 |
|  | a | a | a |  |  |  |
| Total volatile emission (N =154) | 30.69 ± 7.16 | 28.69 ± 2.9 | 31.05 ± 3.27 | 1.35 | 2 | 0.508 |
|  | a | a | a |  |  |  |

**Table S2.** **The effects of temperature- and pollinator-mediated evolution on plant fitness and pollinator preference, in local and foreign temperature environments**. Output of (generalized) linear mixed models showing effects of temperature environment, temperature-mediated evolution and pollination-mediated evolution on plant fitness and pollinator attraction of *Brassica rapa* plants. Replicate and cohort were included as random factors in all models. Bold values indicate results where *P* ≤ 0.05. Underlined values indicate results where *P* ≤ 0.07.

| Trait | Factor | χ^2^ | DF | P |
| --- | --- | --- | --- | --- |
| Flower visits | Temperature environment (E) | 1.87 | 1 | 0.171 |
|  | Temperature genotype (T) | 0.03 | 1 | 0.868 |
|  | Pollination genotype (P) | 0.02 | 1 | 0.894 |
|  | **E x T** | **5.64** | **1** | **0.018** |
|  | E x P | 0.24 | 1 | 0.626 |
|  | T x P | 1.12 | 1 | 0.291 |
|  | E x T x P | 1.01 | 1 | 0.316 |
| Bees per plant | Temperature environment (E) | 0.12 | 1 | 0.729 |
|  | Temperature genotype (T) | 0.07 | 1 | 0.790 |
|  | Pollination genotype (P) | 0.01 | 1 | 0.938 |
|  | E x T | 1.13 | 1 | 0.287 |
|  | E x P | 0.12 | 1 | 0.732 |
|  | T x P | 0.17 | 1 | 0.684 |
|  | E x T x P | 0.01 | 1 | 0.928 |
| Mean seconds per flower | Temperature environment (E) | 1.25 | 1 | 0.263 |
|  | Temperature genotype (T) | 0.18 | 1 | 0.671 |
|  | Pollination genotype (P) | 2.89 | 1 | 0.089 |
|  | E x T | 0.03 | 1 | 0.857 |
|  | E x P | 0.32 | 1 | 0.573 |
|  | T x P | 0.66 | 1 | 0.415 |
|  | E x T x P | 0.20 | 1 | 0.654 |
| Seconds per plant | Temperature environment (E) | 0.41 | 1 | 0.524 |
|  | Temperature genotype (T) | 0.00 | 1 | 0.950 |
|  | Pollination genotype (P) | 0.97 | 1 | 0.325 |
|  | E x T | 3.48 | 1 | 0.062 |
|  | E x P | 1.10 | 1 | 0.293 |
|  | T x P | 0.66 | 1 | 0.418 |
|  | E x T x P | 0.90 | 1 | 0.344 |
| Seeds per plant | Temperature environment (E) | 1.40 | 1 | 0.237 |
|  | Temperature genotype (T) | 1.55 | 1 | 0.214 |
|  | **Pollination genotype (P)** | **15.18** | **1** | **<0.001** |
|  | E x T | 0.17 | 1 | 0.677 |
|  | E x P | 0.00 | 1 | 0.983 |
|  | T x P | 1.96 | 1 | 0.161 |
|  | E x T x P | 0.90 | 1 | 0.342 |
| Fruits per plant | Temperature environment (E) | 0.03 | 1 | 0.868 |
|  | Temperature genotype (T) | 0.00 | 1 | 0.965 |
|  | **Pollination genotype (P)** | **5.96** | **1** | **0.015** |
|  | E x T | 0.59 | 1 | 0.444 |
|  | E x P | 0.07 | 1 | 0.788 |
|  | T x P | 0.18 | 1 | 0.670 |
|  | E x T x P | 2.55 | 1 | 0.111 |
| Seeds per fruit | Temperature environment (E) | 0.63 | 1 | 0.429 |
|  | Temperature genotype (T) | 0.27 | 1 | 0.602 |
|  | Pollination genotype (P) | 0.03 | 1 | 0.862 |
|  | E x T | 0.38 | 1 | 0.536 |
|  | E x P | 1.08 | 1 | 0.300 |
|  | T x P | 0.10 | 1 | 0.757 |
|  | E x T x P | 0.48 | 1 | 0.490 |
| Fruit set | Temperature environment (E) | 2.53 | 1 | 0.112 |
|  | Temperature genotype (T) | 0.53 | 1 | 0.469 |
|  | Pollination genotype (P) | 0.30 | 1 | 0.581 |
|  | E x T | 0.37 | 1 | 0.543 |
|  | E x P | 0.15 | 1 | 0.695 |
|  | T x P | 0.33 | 1 | 0.565 |
|  | E x T x P | 0.11 | 1 | 0.746 |

**Table S3. The interactive effects of temperature environment and temperature evolution on plant fitness and pollinator preference, in bumblebee- and control-genotype plants.** Output of (generalized) linear mixed models showing effects of temperature environment and temperature-mediated evolution on plant fitness and pollinator attraction of *Brassica rapa* plants. Replicate and cohort were included as random factors in all models. Bold values indicate results where *P* ≤ 0.05. Underlined values indicate results where *P* ≤ 0.07.

|  |  | Control | | | Bumblebee-genotype | | |
| --- | --- | --- | --- | --- | --- | --- | --- |
| Trait | Factor | χ^2^ | DF | P | χ^2^ | DF | P |
| Flower visits | Temperature genotype (T) | 0.47 | 1 | 0.495 | 1.01 | 1 | 0.314 |
|  | Temperature environment (E) | 0.26 | 1 | 0.611 | 1.84 | 1 | 0.175 |
|  | **T x E** | 1.10 | 1 | 0.294 | **5.87** | **1** | **0.015** |
| Bees per plant | Temperature genotype (T) | 0.20 | 1 | 0.652 | 0.01 | 1 | 0.922 |
|  | Temperature environment (E) | 0.00 | 1 | 0.980 | 0.23 | 1 | 0.628 |
|  | T x E | 0.65 | 1 | 0.421 | 0.49 | 1 | 0.485 |
| Mean seconds per flower | Temperature genotype (T) | 0.05 | 1 | 0.828 | 0.85 | 1 | 0.356 |
|  | Temperature environment (E) | 1.29 | 1 | 0.255 | 0.11 | 1 | 0.742 |
|  | T x E | 0.02 | 1 | 0.880 | 0.22 | 1 | 0.639 |
| Seconds per plant | Temperature genotype (T) | 0.55 | 1 | 0.457 | 0.58 | 1 | 0.447 |
|  | Temperature environment (E) | 0.04 | 1 | 0.841 | 1.66 | 1 | 0.197 |
|  | **T x E** | 0.60 | 1 | 0.437 | **4.06** | **1** | **0.044** |
| First visits | Temperature genotype (T) | 0.77 | 1 | 0.381 | 0.10 | 1 | 0.752 |
|  | Temperature environment (E) | 0.01 | 1 | 0.920 | 0.07 | 1 | 0.798 |
|  | **T x E** | 0.12 | 1 | 0.733 | **5.11** | **1** | **0.024** |
| Second visits | Temperature genotype (T) | 1.81 | 1 | 0.178 | 0.73 | 1 | 0.394 |
|  | Temperature environment (E) | 0.00 | 1 | 0.965 | 0.10 | 1 | 0.756 |
|  | T x E | 1.93 | 1 | 0.165 | 0.00 | 1 | 0.967 |
| Seeds per plant | Temperature genotype (T) | **4.11** | **1** | **0.043** | 0.00 | 1 | 0.988 |
|  | Temperature environment (E) | 0.80 | 1 | 0.371 | 0.64 | 1 | 0.425 |
|  | T x E | 1.28 | 1 | 0.257 | 0.09 | 1 | 0.761 |
| Fruits per plant | Temperature genotype (T) | 0.09 | 1 | 0.766 | 0.11 | 1 | 0.740 |
|  | Temperature environment (E) | 0.16 | 1 | 0.687 | 0.01 | 1 | 0.926 |
|  | T x E | 3.32 | 1 | 0.069 | 0.19 | 1 | 0.660 |
| Seeds per fruit | Temperature genotype (T) | 0.05 | 1 | 0.826 | 0.89 | 1 | 0.345 |
|  | Temperature environment (E) | 3.36 | 1 | 0.067 | 0.59 | 1 | 0.443 |
|  | T x E | 0.50 | 1 | 0.481 | 0.05 | 1 | 0.820 |
| Fruit set | Temperature genotype (T) | 0.20 | 1 | 0.651 | 0.09 | 1 | 0.766 |
|  | Temperature environment (E) | 0.58 | 1 | 0.447 | 0.16 | 1 | 0.687 |
|  | T x E | 0.84 | 1 | 0.359 | 3.32 | 1 | 0.069 |

**Table S4. Average fitness and pollinator preference for plants tested at their local vs. foreign temperature.** Means ± S.E. of plant fitness and pollinator attraction of *Brassica rapa* plants which for 6 generations were either kept at 23^o^C or at 27°C plus 6 “hot” days of 30^o^C and visited by *Bombus terrestris* bumblebees or hand pollinated (control). On the last generation plants were grown at both their local and foreign temperatures (23^o^C or 27°C plus 6 “hot” days of 30^o^C) and exposed to *Bombus terrestris* bumblebees.

|  |  | Control | | | | Bumblebee-genotype | | | |
| --- | --- | --- | --- | --- | --- | --- | --- | --- | --- |
|  |  | Ambient environment | | Hot environment | | Ambient environment | | Hot environment | |
|  | number | Amb-genotype | Hot-genotype | Amb-genotype | Hot-genotype | Amb-genotype | Hot-genotype | Amb-genotype | Hot-genotype |
| Flower visits | 547 | 26.79 ± 3.56 | 20.83 ± 3.05 | 25.25 ± 4.17 | 26.17 ± 4.22 | 25 ± 3.33 | 19.69 ± 2.45 | 21.05 ± 3.46 | 34.34 ± 5.91 |
|  |  |  |  |  |  |  |  |  |  |
| Bees per plant | 547 | 1.35 ± 0.15 | 1.16 ± 0.13 | 1.23 ± 0.16 | 1.28 ± 0.17 | 1.34 ± 0.15 | 1.25 ± 0.14 | 1.17 ± 0.14 | 1.28 ± 0.17 |
|  |  |  |  |  |  |  |  |  |  |
| Mean seconds per flower | 532 | 5.14 ± 0.61 | 5.2 ± 0.54 | 4.3 ± 0.45 | 4.53 ± 0.7 | 4.28 ± 0.42 | 4.02 ± 0.41 | 4.35 ± 0.65 | 3.58 ± 0.36 |
|  |  |  |  |  |  |  |  |  |  |
| Seconds per plant | 532 | 178.14 ± 25.03 | 142.12 ± 21.26 | 155.43 ± 26.98 | 155.57 ± 26.33 | 144.29 ± 21.14 | 112.84 ± 16.27 | 123.59 ± 22.44 | 185.85 ± 31.66 |
|  |  |  |  |  |  |  |  |  |  |
| First visits | 547 | 0.25 ± 0.06 | 0.29 ± 0.07 | 0.23 ± 0.05 | 0.31 ± 0.08 | 0.38 ± 0.08 | 0.19 ± 0.05 | 0.2 ± 0.05 | 0.33 ± 0.08 |
|  |  |  |  |  |  |  |  |  |  |
| Second visits | 547 | 0.35 ± 0.06 | 0.17 ± 0.05 | 0.27 ± 0.07 | 0.26 ± 0.07 | 0.3 ± 0.08 | 0.24 ± 0.06 | 0.27 ± 0.06 | 0.22 ± 0.06 |
|  |  |  |  |  |  |  |  |  |  |
| Seeds per plant | 544 | 140.69 ± 19.92 | 84.74 ± 16.47 | 103.63 ± 15.84 | 88.8 ± 15.66 | 171.91 ± 26.87 | 181.16 ± 27.43 | 158.2 ± 29.24 | 150.34 ± 25.5 |
|  |  |  |  |  |  |  |  |  |  |
| Fruits per plant | 405 | 9.46 ± 1.63 | 5.72 ± 1.25 | 6.93 ± 1.26 | 8.58 ± 1.93 | 11.00 ± 2.27 | 12.79 ± 2.81 | 10.67 ± 2.30 | 10.80 ± 2.30 |
|  |  |  |  |  |  |  |  |  |  |
| Seeds per fruit | 405 | 11.5 ± 1.14 | 10.97 ± 1.18 | 9.09 ± 0.69 | 9.82 ± 0.99 | 10.63 ± 0.92 | 9.96 ± 0.84 | 11.65 ± 0.94 | 10.47 ± 1.3 |
|  |  |  |  |  |  |  |  |  |  |
| Fruit set | 404 | 0.45 ± 0.07 | 0.33 ± 0.06 | 0.32 ± 0.05 | 0.32 ± 0.06 | 0.4 ± 0.07 | 0.4 ± 0.07 | 0.33 ± 0.06 | 0.32 ± 0.06 |
|  |  |  |  |  |  |  |  |  |  |

**Table S5. Bumblebee preference for plant traits.** Estimates ± standard error and significance (p-values) of generalized linear mixed models testing the relationship between pollinator (*Bombus terrestris*) plant visits and *Brassica rapa* plant traits. Visual and scent traits were modelled separately. Bold values indicate results where *P* ≤ 0.05. Scent emission is expressed as pg / flower / l / h. UV relative area calculated by dividing the UV-absorbing surface of the petal (in cm^2^) by the total petal area (in cm^2^). RDR (relative diffuse reflectance) indicates the relative percentage of reflectance.

| Estimate | VIF | β ± SE | z-value | P-value |
| --- | --- | --- | --- | --- |
| **Plant height (cm)** | **1.21** | **0.11 ± 0.05** | **2.00** | **0.046** |
| **Flower number** | **1.66** | **0.24 ± 0.07** | **3.37** | **0.001** |
| **Leaf number** | **1.25** | **0.14 ± 0.05** | **2.67** | **0.008** |
| **Petal area (cm^2^)** | **1.10** | **0.11 ± 0.05** | **2.34** | **0.020** |
| Nectar | >5.00 | - | - | - |
| Nectar per plant (µl) | 1.57 | -0.05 ± 0.06 | -0.86 | 0.392 |
| *Visual* |  |  |  |  |
| UV relative area (ratio) | 1.79 | 0.00 ± 0.06 | 0.08 | 0.936 |
| UVA RDR (%) | 3.78 | -0.01 ± 0.09 | -0.12 | 0.901 |
| UVB RDR (%) | 4.86 | 0.03 ± 0.10 | 0.33 | 0.738 |
| Yellow (%) | 1.82 | 0.05 ± 0.06 | 0.80 | 0.422 |
| *Scent* |  |  |  |  |
| Benzaldehyde | 1.44 | -0.08 ± 0.05 | -1.47 | 0.141 |
| 1-butene-4-isothyocyanate | 1.19 | -0.06 ± 0.05 | -1.21 | 0.227 |
| Methyl benzoate | 1.94 | 0.04 ± 0.06 | 0.73 | 0.466 |
| Phenylethyl alcohol | 3.88 | -0.01 ± 0.08 | -0.11 | 0.916 |
| 2-Amino benzaldehyde | 4.35 | 0.01 ± 0.09 | 0.14 | 0.891 |
| p-Anisaldehyde | 1.40 | -0.02 ± 0.05 | -0.45 | 0.652 |
| Methyl anthranilate | 3.33 | 0.00 ± 0.08 | 0.00 | 0.998 |
| *(Z)*-3-Hexen-1-ol acetate | 1.17 | -0.09 ± 0.05 | -1.79 | 0.073 |
| Phenylacetaldehyde | 4.05 | 0.01 ± 0.09 | 0.09 | 0.929 |
| Benzyl nitrile | 3.94 | -0.05 ± 0.09 | -0.57 | 0.570 |
| Methyl salicilate | 1.59 | 0.03 ± 0.05 | 0.63 | 0.529 |
| Indole | 4.10 | 0.04 ± 0.09 | 0.46 | 0.643 |
| *(E,E)*-α-Farnesene | 1.88 | 0.06 ± 0.06 | 0.93 | 0.351 |

**Table S6. The effects of temperature-mediated plasticity, temperature-mediated evolution and pollinator-mediated evolution on plant traits.** Average trait values for plants evolved at two temperatures, pollination genotype treatments, and grown at local and foreign temperatures. Scent emission is expressed as pg / flower / l / h. UV relative area calculated by dividing the UV-absorbing surface of the petal (in cm^2^) by the total petal area (in cm^2^). RDR (relative diffuse reflectance) indicates the relative percentage of reflectance.

|  |  | Control | | | | Bumblebee-genotype | | | |
| --- | --- | --- | --- | --- | --- | --- | --- | --- | --- |
|  |  | Ambient environment | | Hot environment | | Ambient environment | | Hot environment | |
|  | number | Amb-genotype | Hot-genotype | Amb-genotype | Hot-genotype | Amb-genotype | Hot-genotype | Amb-genotype | Hot-genotype |
| Height (cm) | 558 | 37.00 ± 0.67 | 35.89 ± 0.70 | 39.31 ± 0.92 | 39.97 ± 1.00 | 36.16 ± 0.82 | 34.88 ± 0.75 | 40.85 ± 1.15 | 39.26 ± 1.02 |
|  |  |  |  |  |  |  |  |  |  |
| Leaf Number | 558 | 9.52 ± 0.30 | 8.68 ± 0.34 | 11.70 ± 0.51 | 10.56 ± 0.36 | 8.29 ± 0.25 | 9.33 ± 0.30 | 11.04 ± 0.37 | 11.64 ± 0.40 |
|  |  |  |  |  |  |  |  |  |  |
| Flower number | 558 | 14.27 ± 0.87 | 12.10 ± 0.77 | 18.70 ± 1.35 | 16.24 ± 1.09 | 15.79 ± 0.95 | 14.81 ± 0.81 | 21.63 ± 1.29 | 22.67 ± 1.58 |
|  |  |  |  |  |  |  |  |  |  |
| Nectar (µl) | 502 | 0.08 ± 0.01 | 0.06 ± 0.01 | 0.08 ± 0.01 | 0.07 ± 0.01 | 0.05 ± 0.01 | 0.05 ± 0.01 | 0.05 ± 0.01 | 0.07 ± 0.01 |
|  |  |  |  |  |  |  |  |  |  |
| Nectar per plant (µl) | 437 | 1.23 ± 0.13 | 0.89 ± 0.09 | 1.62 ± 0.21 | 1.38 ± 0.14 | 1.06 ± 0.15 | 0.96 ± 0.09 | 1.44 ± 0.15 | 1.95 ± 0.25 |
|  |  |  |  |  |  |  |  |  |  |
| Days to flower | 560 | 19.39 ± 0.16 | 19.03 ± 0.13 | 17.44 ± 0.10 | 17.6 ± 0.12 | 19.53 ± 0.15 | 19.49 ± 0.12 | 17.97 ± 0.17 | 17.74 ± 0.14 |
|  |  |  |  |  |  |  |  |  |  |
| Petal area (cm^2^) | 506 | 0.25 ± 0.01 | 0.26 ± 0.00 | 0.19 ± 0.00 | 0.20 ± 0.00 | 0.23 ± 0.01 | 0.23 ± 0.01 | 0.19 ± 0.01 | 0.18 ± 0.00 |
|  |  |  |  |  |  |  |  |  |  |
| UV relative area (ratio) | 506 | 0.30 ± 0.01 | 0.31 ± 0.02 | 0.28 ± 0.01 | 0.31 ± 0.02 | 0.27 ± 0.01 | 0.25 ± 0.02 | 0.28 ± 0.01 | 0.23 ± 0.01 |
|  |  |  |  |  |  |  |  |  |  |
| UVA RDR (%) | 507 | 73.04 ± 2.11 | 68.43 ± 1.87 | 75.80 ± 1.35 | 67.24 ± 2.33 | 72.48 ± 1.80 | 77.35 ± 2.12 | 73.37 ± 1.89 | 77.15 ± 1.78 |
|  |  |  |  |  |  |  |  |  |  |
| UVB RDR (%) | 507 | 70.93 ± 1.92 | 71.30 ± 1.71 | 73.20 ± 1.28 | 69.55 ± 2.29 | 71.48 ± 1.60 | 75.41 ± 2.13 | 71.48 ± 1.80 | 75.66 ± 1.68 |
|  |  |  |  |  |  |  |  |  |  |
| Yellow (%) | 507 | 81.96 ± 1.11 | 80.63 ± 1.14 | 82.38 ± 0.98 | 81.53 ± 0.97 | 80.88 ± 0.94 | 82.98 ± 1.03 | 83.35 ± 1.12 | 83.47 ± 1.10 |
|  |  |  |  |  |  |  |  |  |  |
| Benzaldehyde | 496 | 3.15 ± 0.39 | 3.01 ± 0.25 | 2.61 ± 0.20 | 2.69 ± 0.24 | 2.33 ± 0.17 | 2.93 ± 0.34 | 2.42 ± 0.25 | 2.58 ± 0.39 |
|  |  |  |  |  |  |  |  |  |  |
| 1-Butene-4-isothyocyanate | 496 | 0.52 ± 0.07 | 0.99 ± 0.15 | 2.14 ± 0.48 | 2.53 ± 0.42 | 0.55 ± 0.09 | 0.51 ± 0.12 | 1.49 ± 0.26 | 1.24 ± 0.28 |
|  |  |  |  |  |  |  |  |  |  |
| Methyl Benzoate | 496 | 0.55 ± 0.10 | 0.42 ± 0.07 | 0.35 ± 0.03 | 0.36 ± 0.04 | 0.25 ± 0.04 | 0.22 ± 0.03 | 0.20 ± 0.02 | 0.18 ± 0.02 |
|  |  |  |  |  |  |  |  |  |  |
| Phenylethyl alcohol | 496 | 0.07 ± 0.01 | 0.07 ± 0.01 | 0.05 ± 0.01 | 0.04 ± 0.00 | 0.06 ± 0.02 | 0.05 ± 0.01 | 0.03 ± 0.00 | 0.03 ± 0.01 |
|  |  |  |  |  |  |  |  |  |  |
| 2-Amino benzaldehyde | 496 | 9.91 ± 1.53 | 11.30 ± 1.69 | 6.24 ± 1.35 | 7.59 ± 1.31 | 6.04 ± 0.85 | 9.35 ± 1.47 | 6.26 ± 1.22 | 4.24 ± 0.67 |
|  |  |  |  |  |  |  |  |  |  |
| p-Anisaldehyde | 496 | 0.10 ± 0.02 | 0.09 ± 0.02 | 0.05 ± 0.01 | 0.06 ± 0.01 | 0.18 ± 0.03 | 0.07 ± 0.01 | 0.12 ± 0.02 | 0.04 ± 0.01 |
|  |  |  |  |  |  |  |  |  |  |
| Methyl anthranilate | 496 | 1.30 ± 0.22 | 0.93 ± 0.17 | 0.85 ± 0.23 | 0.71 ± 0.19 | 0.54 ± 0.15 | 1.04 ± 0.16 | 0.43 ± 0.08 | 0.51 ± 0.10 |
|  |  |  |  |  |  |  |  |  |  |
| *(Z)*-3-Hexen-1-ol acetate | 496 | 0.54 ± 0.09 | 0.56 ± 0.08 | 0.73 ± 0.09 | 0.56 ± 0.06 | 0.31 ± 0.06 | 0.29 ± 0.05 | 0.49 ± 0.08 | 0.53 ± 0.09 |
|  |  |  |  |  |  |  |  |  |  |
| Phenylacetaldehyde | 496 | 0.22 ± 0.06 | 0.22 ± 0.05 | 0.06 ± 0.01 | 0.05 ± 0.01 | 0.19 ± 0.08 | 0.10 ± 0.02 | 0.04 ± 0.01 | 0.03 ± 0.01 |
|  |  |  |  |  |  |  |  |  |  |
| Benzyl nitrile | 496 | 0.95 ± 0.14 | 1.01 ± 0.17 | 0.31 ± 0.05 | 0.35 ± 0.05 | 0.70 ± 0.11 | 0.72 ± 0.08 | 0.31 ± 0.05 | 0.24 ± 0.04 |
|  |  |  |  |  |  |  |  |  |  |
| Methyl salicilate | 496 | 0.38 ± 0.04 | 0.19 ± 0.02 | 0.23 ± 0.02 | 0.19 ± 0.02 | 0.26 ± 0.04 | 0.27 ± 0.03 | 0.19 ± 0.02 | 0.18 ± 0.02 |
|  |  |  |  |  |  |  |  |  |  |
| Indole | 496 | 2.64 ± 0.47 | 2.89 ± 0.56 | 1.65 ± 0.45 | 1.60 ± 0.20 | 2.01 ± 0.34 | 2.36 ± 0.4 | 2.01 ± 0.51 | 1.14 ± 0.24 |
|  |  |  |  |  |  |  |  |  |  |
| *(E,E)*-α-Farnesene | 496 | 8.36 ± 0.75 | 9.37 ± 0.85 | 5.36 ± 0.75 | 7.15 ± 0.70 | 7.83 ± 0.89 | 11.04 ± 1.07 | 6.08 ± 0.77 | 5.55 ± 0.74 |
|  |  |  |  |  |  |  |  |  |  |
| Total volatile emission | 496 | 28.69 ± 2.90 | 31.05 ± 3.27 | 20.63 ± 3.03 | 23.86 ± 2.46 | 21.26 ± 2.26 | 28.94 ± 3.12 | 20.08 ± 2.57 | 16.48 ± 1.60 |

**Table S7.** **Plant trait divergence in response to temperature environment, temperature-mediated evolution, and pollination-mediated evolution.** Output of (generalized) linear mixed models performed on the measured traits of *Brassica rapa* plants. Plants of generation one were not included in this analysis. Replicate and cohort were included as random factors in all models. Bold values indicate results where *P* ≤ 0.05. Underlined values indicate results where *P* ≤ 0.07. “Effect” denotes the trends for bumblebee- and/or hot-evolved and/or hot-environment plants (+ increase; - decrease; = no change) in either plasticity or evolution. For the interactions, effects are only given for significant TxP interactions, as they represent evolutionary effects (A: ambient; H: hot). Scent emission is expressed as pg / flower / l / h. UV relative area calculated by dividing the UV-absorbing surface of the petal (in cm^2^) by the total petal area (in cm^2^). RDR (relative diffuse reflectance) indicates the relative percentage of reflectance.

|  |  | All treatments | | | | Bumblebee-genotype | | | | Control | | | |
| --- | --- | --- | --- | --- | --- | --- | --- | --- | --- | --- | --- | --- | --- |
| Trait | Factor | χ^2^ | DF | P | Effect | χ^2^ | DF | P | Effect | χ^2^ | DF | P | Effect |
| Height | Temperature environment (E) | **40.09** | **1** | **<0.001** | **+** | **25.13** | **1** | **<0.001** | **+** | **15.5** | **1** | **<0.001** | **+** |
|  | Temperature genotype (T) | 2.23 | 1 | 0.135 |  | 3.14 | 1 | 0.076 |  | 0.08 | 1 | 0.783 |  |
|  | Pollination genotype (P) | 0.27 | 1 | 0.606 |  |  |  |  |  |  |  |  |  |
|  | E x T | 0.42 | 1 | 0.519 |  | 0.01 | 1 | 0.916 |  | 1.18 | 1 | 0.278 |  |
|  | E x P | 1.19 | 1 | 0.276 |  |  |  |  |  |  |  |  |  |
|  | T x P | 1.20 | 1 | 0.273 |  |  |  |  |  |  |  |  |  |
|  | E x T x P | 0.66 | 1 | 0.415 |  |  |  |  |  |  |  |  |  |
| Flower number | Temperature environment (E) | **51.34** | **1** | **<0.001** | **+** | **34.07** | **1** | **<0.001** | **+** | **18.49** | **1** | **<0.001** | **+** |
|  | Temperature genotype (T) | 2.51 | 1 | 0.113 |  | 0.00 | 1 | 0.971 |  | **5.03** | **1** | **0.025** | **-** |
|  | Pollination genotype (P) | **19.05** | **1** | **<0.001** | **+** |  |  |  |  |  |  |  |  |
|  | E x T | 0.57 | 1 | 0.448 |  | 0.79 | 1 | 0.375 |  | 0.04 | 1 | 0.846 |  |
|  | E x P | 0.91 | 1 | 0.341 |  |  |  |  |  |  |  |  |  |
|  | T x P | 2.68 | 1 | 0.101 |  |  |  |  |  |  |  |  |  |
|  | E x T x P | 0.23 | 1 | 0.632 |  |  |  |  |  |  |  |  |  |
| Leaf number | Temperature environment (E) | **79.23** | **1** | **<0.001** | **+** | **60.90** | **1** | **<0.001** | **+** | **31.9** | **1** | **<0.001** | **+** |
|  | Temperature genotype (T) | 0.17 | 1 | 0.680 |  | **6.55** | **1** | **0.011** | **+** | **7.35** | **1** | **0.010** | **-** |
|  | Pollination genotype (P) | 0.04 | 1 | 0.835 |  |  |  |  |  |  |  |  |  |
|  | E x T | 0.54 | 1 | 0.462 |  | 0.92 | 1 | 0.337 |  | 0.02 | 1 | 0.880 |  |
|  | E x P | 1.11 | 1 | 0.293 |  |  |  |  |  |  |  |  |  |
|  | T x P | **12.26** | **1** | **<0.001** | **A- H+** |  |  |  |  |  |  |  |  |
|  | E x T x P | 0.29 | 1 | 0.587 |  |  |  |  |  |  |  |  |  |
| Petal area | Temperature environment (E) | **251.65** | **1** | **<0.001** | **-** | **107.80** | **1** | **<0.001** | **-** | **163** | **1** | **<0.001** | **-** |
|  | Temperature genotype (T) | 0.00 | 1 | 0.952 |  | 1.99 | 1 | 0.158 |  | 1.29 | 1 | 0.260 |  |
|  | Pollination genotype (P) | **21.90** | **1** | **<0.001** | **-** |  |  |  |  |  |  |  |  |
|  | E x T | 0.16 | 1 | 0.691 |  | 1.23 | 1 | 0.268 |  | 0.31 | 1 | 0.580 |  |
|  | E x P | 2.31 | 1 | 0.128 |  |  |  |  |  |  |  |  |  |
|  | T x P | 2.52 | 1 | 0.113 |  |  |  |  |  |  |  |  |  |
|  | E x T x P | 1.31 | 1 | 0.253 |  |  |  |  |  |  |  |  |  |
| Nectar per plant | Temperature environment (E) | **27.48** | **1** | **<0.001** | **+** | **16.89** | **1** | **<0.001** | **+** | **9.85** | **1** | **<0.001** | **+** |
|  | Temperature genotype (T) | 0.80 | 1 | 0.370 |  | 0.78 | 1 | 0.377 |  | **4.34** | **1** | **0.040** | **-** |
|  | Pollination genotype (P) | 0.16 | 1 | 0.693 |  |  |  |  |  |  |  |  |  |
|  | E x T | 2.93 | 1 | 0.087 |  | 2.93 | 1 | 0.087 |  | 0.38 | 1 | 0.540 |  |
|  | E x P | 0.89 | 1 | 0.347 |  |  |  |  |  |  |  |  |  |
|  | T x P | **4.40** | **1** | **0.036** | **A- H+** |  |  |  |  |  |  |  |  |
|  | E x T x P | 0.62 | 1 | 0.433 |  |  |  |  |  |  |  |  |  |
| Nectar per flower | Temperature environment (E) | 1.27 | 1 | 0.259 |  | 0.84 | 1 | 0.360 |  | 0.4 | 1 | 0.530 |  |
|  | Temperature genotype (T) | 0.13 | 1 | 0.719 |  | 1.61 | 1 | 0.204 |  | 3.01 | 1 | 0.080 |  |
|  | Pollination genotype (P) | **19.02** | **1** | **<0.001** | **-** |  |  |  |  |  |  |  |  |
|  | E x T | 1.31 | 1 | 0.253 |  | 1.85 | 1 | 0.174 |  | 0.08 | 1 | 0.780 |  |
|  | E x P | 0.05 | 1 | 0.815 |  |  |  |  |  |  |  |  |  |
|  | T x P | **4.62** | **1** | **0.032** | **A- H-** |  |  |  |  |  |  |  |  |
|  | E x T x P | 0.73 | 1 | 0.393 |  |  |  |  |  |  |  |  |  |
| Days until flowering | Temperature environment (E) | **190.38** | **1** | **<0.001** | **-** | **70.86** | **1** | **<0.001** | **-** | **126** | **1** | **<0.001** | **-** |
|  | Temperature genotype (T) | 1.54 | 1 | 0.215 |  | 2.14 | 1 | 0.144 |  | 0.01 | 1 | 0.900 |  |
|  | Pollination genotype (P) | **18.40** | **1** | **<0.001** | **+** |  |  |  |  |  |  |  |  |
|  | E x T | 0.54 | 1 | 0.464 |  | 1.30 | 1 | 0.253 |  | **7.46** | **1** | **0.010** | **A- H=** |
|  | E x P | **4.50** | **1** | **0.034** | **C- B-** |  |  |  |  |  |  |  |  |
|  | T x P | 1.91 | 1 | 0.167 |  |  |  |  |  |  |  |  |  |
|  | E x T x P | **7.99** | **1** | **0.005** |  |  |  |  |  |  |  |  |  |
| UV relative area | Temperature environment (E) | 0.61 | 1 | 0.436 |  | 0.06 | 1 | 0.805 |  | 0.35 | 1 | 0.553 |  |
|  | Temperature genotype (T) | 2.66 | 1 | 0.103 |  | **11.09** | **1** | **0.001** | **-** | 1.13 | 1 | 0.287 |  |
|  | Pollination genotype (P) | **21.47** | **1** | **<0.001** | **-** |  |  |  |  |  |  |  |  |
|  | E x T | 0.01 | 1 | 0.914 |  | 0.97 | 1 | 0.324 |  | 0.81 | 1 | 0.369 |  |
|  | E x P | 0.10 | 1 | 0.751 |  |  |  |  |  |  |  |  |  |
|  | T x P | **10.64** | **1** | **0.001** | **A= H-** |  |  |  |  |  |  |  |  |
|  | E x T x P | 2.41 | 1 | 0.121 |  |  |  |  |  |  |  |  |  |
| UVA RDR | Temperature environment (E) | 0.15 | 1 | 0.695 |  | 0.01 | 1 | 0.919 |  | 0.17 | 1 | 0.683 |  |
|  | Temperature genotype (T) | 1.24 | 1 | 0.266 |  | **4.12** | **1** | **0.042** | **+** | **11.58** | **1** | **0.001** | **-** |
|  | Pollination genotype (P) | **7.47** | **1** | **0.006** | **+** |  |  |  |  |  |  |  |  |
|  | E x T | 0.98 | 1 | 0.323 |  | 0.11 | 1 | 0.741 |  | 1.03 | 1 | 0.310 |  |
|  | E x P | 0.05 | 1 | 0.824 |  |  |  |  |  |  |  |  |  |
|  | T x P | **15.52** | **1** | **<0.001** | **A= H+** |  |  |  |  |  |  |  |  |
|  | E x T x P | 0.26 | 1 | 0.613 |  |  |  |  |  |  |  |  |  |
| UVB RDR | Temperature environment (E) | 0.02 | 1 | 0.902 |  | 0.00 | 1 | 0.994 |  | 0.02 | 1 | 0.882 |  |
|  | Temperature genotype (T) | 0.68 | 1 | 0.409 |  | **4.74** | **1** | **0.030** | **+** | 0.82 | 1 | 0.364 |  |
|  | Pollination genotype (P) | 2.80 | 1 | 0.094 |  |  |  |  |  |  |  |  |  |
|  | E x T | 0.66 | 1 | 0.416 |  | 0.01 | 1 | 0.923 |  | 1.22 | 1 | 0.270 |  |
|  | E x P | 0.01 | 1 | 0.917 |  |  |  |  |  |  |  |  |  |
|  | T x P | **4.79** | **1** | **0.029** | **A= H+** |  |  |  |  |  |  |  |  |
|  | E x T x P | 0.77 | 1 | 0.381 |  |  |  |  |  |  |  |  |  |
| Yellow | Temperature environment (E) | 2.10 | 1 | 0.147 |  | 2.05 | 1 | 0.152 |  | 0.4 | 1 | 0.530 |  |
|  | Temperature genotype (T) | 0.03 | 1 | 0.864 |  | 0.99 | 1 | 0.321 |  | 1.3 | 1 | 0.260 |  |
|  | Pollination genotype (P) | 1.79 | 1 | 0.181 |  |  |  |  |  |  |  |  |  |
|  | E x T | 0.36 | 1 | 0.546 |  | 1.01 | 1 | 0.315 |  | 0.05 | 1 | 0.830 |  |
|  | E x P | 0.32 | 1 | 0.574 |  |  |  |  |  |  |  |  |  |
|  | T x P | 2.13 | 1 | 0.144 |  |  |  |  |  |  |  |  |  |
|  | E x T x P | 0.67 | 1 | 0.412 |  |  |  |  |  |  |  |  |  |
| Benzaldehyde | Temperature environment (E) | 2.54 | 1 | 0.111 |  | 1.06 | 1 | 0.304 |  | 1.61 | 1 | 0.200 |  |
|  | Temperature genotype (T) | 0.84 | 1 | 0.359 |  | 0.96 | 1 | 0.327 |  | 0.02 | 1 | 0.890 |  |
|  | Pollination genotype (P) | **3.88** | **1** | **0.049** | **-** |  |  |  |  |  |  |  |  |
|  | E x T | 0.60 | 1 | 0.440 |  | 0.63 | 1 | 0.427 |  | 0.09 | 1 | 0.770 |  |
|  | E x P | 0.02 | 1 | 0.900 |  |  |  |  |  |  |  |  |  |
|  | T x P | 0.52 | 1 | 0.471 |  |  |  |  |  |  |  |  |  |
|  | E x T x P | 0.14 | 1 | 0.710 |  |  |  |  |  |  |  |  |  |
| 1-butene-4-isothyocyanate | Temperature environment (E) | **119.46** | **1** | **<0.001** | **+** | **46.31** | **1** | **<0.001** | **+** | **79.13** | **1** | **<0.001** | **+** |
|  | Temperature genotype (T) | 1.26 | 1 | 0.261 |  | 1.73 | 1 | 0.188 |  | **12.73** | **1** | **<0.001** | **+** |
|  | Pollination genotype (P) | **20.27** | **1** | **<0.001** | **-** |  |  |  |  |  |  |  |  |
|  | E x T | 1.14 | 1 | 0.286 |  | 0.04 | 1 | 0.834 |  | 2.24 | 1 | 0.135 |  |
|  | E x P | 0.11 | 1 | 0.745 |  |  |  |  |  |  |  |  |  |
|  | T x P | **12.97** | **1** | **<0.001** | **A= H-** |  |  |  |  |  |  |  |  |
|  | E x T x P | 0.63 | 1 | 0.426 |  |  |  |  |  |  |  |  |  |
| Methyl benzoate | Temperature environment (E) | **5.13** | **1** | **0.024** | **-** | 3.23 | 1 | 0.072 |  | 2.15 | 1 | 0.143 |  |
|  | Temperature genotype (T) | 2.04 | 1 | 0.153 |  | 0.53 | 1 | 0.468 |  | 2.31 | 1 | 0.129 |  |
|  | Pollination genotype (P) | **83.52** | **1** | **<0.001** | **-** |  |  |  |  |  |  |  |  |
|  | E x T | 3.18 | 1 | 0.075 |  | 0.46 | 1 | 0.497 |  | 3.50 | 1 | 0.061 |  |
|  | E x P | 0.02 | 1 | 0.882 |  |  |  |  |  |  |  |  |  |
|  | T x P | 0.25 | 1 | 0.620 |  |  |  |  |  |  |  |  |  |
|  | E x T x P | 0.56 | 1 | 0.453 |  |  |  |  |  |  |  |  |  |
| Phenylethyl alcohol | Temperature environment (E) | **60.30** | **1** | **<0.001** | **-** | **34.48** | **1** | **<0.001** | **-** | **25.7** | **1** | **<0.001** | **-** |
|  | Temperature genotype (T) | 0.27 | 1 | 0.602 |  | 0.87 | 1 | 0.350 |  | 0.03 | 1 | 0.870 |  |
|  | Pollination genotype (P) | **18.08** | **1** | **<0.001** | **-** |  |  |  |  |  |  |  |  |
|  | E x T | 2.55 | 1 | 0.111 |  | 0.82 | 1 | 0.365 |  | 2.02 | 1 | 0.160 |  |
|  | E x P | 0.11 | 1 | 0.737 |  |  |  |  |  |  |  |  |  |
|  | T x P | 0.40 | 1 | 0.530 |  |  |  |  |  |  |  |  |  |
|  | E x T x P | 0.43 | 1 | 0.514 |  |  |  |  |  |  |  |  |  |
| 2-Amino benzaldehyde | Temperature environment (E) | **22.97** | **1** | **<0.001** | **-** | **13.12** | **1** | **<0.001** | **-** | **11.1** | **1** | **<0.001** | **-** |
|  | Temperature genotype (T) | 2.90 | 1 | 0.089 |  | 0.03 | 1 | 0.854 |  | 3.49 | 1 | 0.060 |  |
|  | Pollination genotype (P) | **6.81** | **1** | **0.009** | **-** |  |  |  |  |  |  |  |  |
|  | E x T | 0.96 | 1 | 0.327 |  | **8.21** | **1** | **0.004** | **A+ H=** | 1.07 | 1 | 0.300 |  |
|  | E x P | 0.01 | 1 | 0.942 |  |  |  |  |  |  |  |  |  |
|  | T x P | 1.08 | 1 | 0.299 |  |  |  |  |  |  |  |  |  |
|  | E x T x P | **7.21** | **1** | **0.007** |  |  |  |  |  |  |  |  |  |
| p-Anisaldehyde | Temperature environment (E) | **30.72** | **1** | **<0.001** | **-** | **13.30** | **1** | **<0.001** | **-** | **14.67** | **1** | **<0.001** | **-** |
|  | Temperature genotype (T) | **18.71** | **1** | **<0.001** | **-** | **57.14** | **1** | **<0.001** | **-** | 0.89 | 1 | 0.347 |  |
|  | Pollination genotype (P) | 2.17 | 1 | 0.141 |  |  |  |  |  |  |  |  |  |
|  | E x T | 1.27 | 1 | 0.260 |  | 1.94 | 1 | 0.163 |  | 0.02 | 1 | 0.891 |  |
|  | E x P | 0.12 | 1 | 0.726 |  |  |  |  |  |  |  |  |  |
|  | T x P | **42.76** | **1** | **<0.001** | **C= B-** |  |  |  |  |  |  |  |  |
|  | E x T x P | 0.54 | 1 | 0.463 |  |  |  |  |  |  |  |  |  |
| Methyl anthranilate | Temperature environment (E) | **20.21** | **1** | **<0.001** | **-** | **10.61** | **1** | **0.001** | **-** | **12.45** | **1** | **<0.001** | **-** |
|  | Temperature genotype (T) | **3.85** | **1** | **0.050** | **+** | **7.39** | **1** | **0.007** | **+** | 0.44 | 1 | 0.507 |  |
|  | Pollination genotype (P) | **21.44** | **1** | **<0.001** | **-** |  |  |  |  |  |  |  |  |
|  | E x T | 0.57 | 1 | 0.452 |  | 3.81 | 1 | 0.051 |  | 0.37 | 1 | 0.546 |  |
|  | E x P | 0.02 | 1 | 0.891 |  |  |  |  |  |  |  |  |  |
|  | T x P | **3.60** | **1** | **0.058** |  |  |  |  |  |  |  |  |  |
|  | E x T x P | 3.26 | 1 | 0.071 |  |  |  |  |  |  |  |  |  |
| *(Z)*-3-Hexen-1-ol acetate | Temperature environment (E) | **20.99** | **1** | **<0.001** | **+** | **15.86** | **1** | **<0.001** | **+** | **4.79** | **1** | **0.029** | **+** |
|  | Temperature genotype (T) | 0.22 | 1 | 0.638 |  | 0.24 | 1 | 0.626 |  | 0.01 | 1 | 0.922 |  |
|  | Pollination genotype (P) | **21.84** | **1** | **<0.001** | **-** |  |  |  |  |  |  |  |  |
|  | E x T | 1.04 | 1 | 0.307 |  | 0.05 | 1 | 0.817 |  | 1.91 | 1 | 0.167 |  |
|  | E x P | 2.87 | 1 | 0.090 |  |  |  |  |  |  |  |  |  |
|  | T x P | 0.23 | 1 | 0.632 |  |  |  |  |  |  |  |  |  |
|  | E x T x P | 0.74 | 1 | 0.391 |  |  |  |  |  |  |  |  |  |
| Phenylacetaldehyde | Temperature environment (E) | **157.91** | **1** | **<0.001** | **-** | **70.57** | **1** | **<0.001** | **-** | **91.34** | **1** | **<0.001** | **-** |
|  | Temperature genotype (T) | 0.50 | 1 | 0.478 |  | **4.12** | **1** | **0.042** | **-** | 1.25 | 1 | 0.263 |  |
|  | Pollination genotype (P) | **22.03** | **1** | **<0.001** | **-** |  |  |  |  |  |  |  |  |
|  | E x T | 0.86 | 1 | 0.353 |  | 0.20 | 1 | 0.655 |  | 0.96 | 1 | 0.328 |  |
|  | E x P | 0.00 | 1 | 0.991 |  |  |  |  |  |  |  |  |  |
|  | T x P | **4.65** | **1** | **0.031** | **-** |  |  |  |  |  |  |  |  |
|  | E x T x P | 0.07 | 1 | 0.796 |  |  |  |  |  |  |  |  |  |
| Benzyl nitrile | Temperature environment (E) | **139.67** | **1** | **<0.001** | **-** | **74.30** | **1** | **<0.001** | **-** | **75.52** | **1** | **<0.001** | **-** |
|  | Temperature genotype (T) | 0.00 | 1 | 0.975 |  | 2.51 | 1 | 0.113 |  | 1.65 | 1 | 0.199 |  |
|  | Pollination genotype (P) | **7.64** | **1** | **0.006** | **-** |  |  |  |  |  |  |  |  |
|  | E x T | 0.55 | 1 | 0.456 |  | 3.62 | 1 | 0.057 |  | 0.45 | 1 | 0.503 |  |
|  | E x P | 0.20 | 1 | 0.653 |  |  |  |  |  |  |  |  |  |
|  | T x P | **3.33** | **1** | **0.068** |  |  |  |  |  |  |  |  |  |
|  | E x T x P | 2.99 | 1 | 0.084 |  |  |  |  |  |  |  |  |  |
| Methyl salicilate | Temperature environment (E) | **18.56** | **1** | **<0.001** | **-** | **11.43** | **1** | **0.001** | **-** | **6.74** | **1** | **0.009** | **-** |
|  | Temperature genotype (T) | **9.35** | **1** | **0.002** | **-** | 0.03 | 1 | 0.873 |  | **19.54** | **1** | **<0.001** | **-** |
|  | Pollination genotype (P) | 0.50 | 1 | 0.480 |  |  |  |  |  |  |  |  |  |
|  | E x T | 1.84 | 1 | 0.175 |  | 0.34 | 1 | 0.558 |  | **6.16** | **1** | **0.013** | **A- H=** |
|  | E x P | 0.41 | 1 | 0.523 |  |  |  |  |  |  |  |  |  |
|  | T x P | **8.01** | **1** | 0.005 | **C- B=** |  |  |  |  |  |  |  |  |
|  | E x T x P | **3.98** | **1** | 0.046 |  |  |  |  |  |  |  |  |  |
| Indole | Temperature environment (E) | **28.61** | **1** | 0.000 | - | **15.58** | **1** | **<0.001** | - | **14.4** | **1** | **<0.001** | **-** |
|  | Temperature genotype (T) | 1.04 | 1 | 0.308 |  | 0.97 | 1 | 0.325 |  | **4.31** | **1** | **0.040** | **+** |
|  | Pollination genotype (P) | 1.83 | 1 | 0.177 |  |  |  |  |  |  |  |  |  |
|  | E x T | 1.82 | 1 | 0.177 |  | **5.55** | **1** | **0.019** | **A= H-** | 0.05 | 1 | 0.820 |  |
|  | E x P | 0.05 | 1 | 0.826 |  |  |  |  |  |  |  |  |  |
|  | T x P | **4.03** | **1** | **0.045** | **A= H-** |  |  |  |  |  |  |  |  |
|  | E x T x P | 3.38 | 1 | 0.066 |  |  |  |  |  |  |  |  |  |
| *(E,E)*-α-Farnesene | Temperature environment (E) | **45.27** | **1** | **<0.001** | **-** | **26.50** | **1** | **<0.001** | **-** | **19.7** | **1** | **<0.001** | **-** |
|  | Temperature genotype (T) | **9.43** | **1** | **0.002** | **+** | 3.02 | 1 | 0.082 |  | **6.61** | **1** | **0.010** | **+** |
|  | Pollination genotype (P) | 0.45 | 1 | 0.501 |  |  |  |  |  |  |  |  |  |
|  | E x T | 0.57 | 1 | 0.451 |  | **5.42** | **1** | **0.020** | **A+ H=** | 1.65 | 1 | 0.200 |  |
|  | E x P | 0.68 | 1 | 0.410 |  |  |  |  |  |  |  |  |  |
|  | T x P | 0.14 | 1 | 0.710 |  |  |  |  |  |  |  |  |  |
|  | E x T x P | **6.70** | **1** | **0.010** |  |  |  |  |  |  |  |  |  |
| Total volatile emission | Temperature environment (E) | **27.74** | **1** | **<0.001** | **-** | **16.20** | **1** | **<0.001** | - | **12.8** | **1** | **<0.001** | **-** |
|  | Temperature genotype (T) | **5.56** | **1** | **0.018** | **+** | 0.99 | 1 | 0.319 |  | **4.47** | **1** | **0.040** | **+** |
|  | Pollination genotype (P) | **9.50** | **1** | **0.002** | **-** |  |  |  |  |  |  |  |  |
|  | E x T | 1.14 | 1 | 0.286 |  | **6.76** | **1** | **0.009** | **A+ H=** | 0.72 | 1 | 0.400 |  |
|  | E x P | 0.10 | 1 | 0.754 |  |  |  |  |  |  |  |  |  |
|  | T x P | 0.36 | 1 | 0.549 |  |  |  |  |  |  |  |  |  |
|  | E x T x P | **6.01** | **1** | **0.014** |  |  |  |  |  |  |  |  |  |
| Sum of aromatic compounds | Temperature environment (E) | **46.65** | **1** | **<0.001** | **-** | **25.79** | **1** | **<0.001** | **-** | **23.24** | **1** | **<0.001** | **-** |
|  | Temperature genotype (T) | 0.37 | 1 | 0.542 |  | 0.33 | 1 | 0.563 |  | 1.09 | 1 | 0.296 |  |
|  | Pollination genotype (P) | **11.01** | **1** | **0.001** | **-** |  |  |  |  |  |  |  |  |
|  | E x T | 0.54 | 1 | 0.463 |  | **5.83** | **1** | **0.016** | **A= H-** | 1.15 | 1 | 0.284 |  |
|  | E x P | 0.04 | 1 | 0.836 |  |  |  |  |  |  |  |  |  |
|  | T x P | 1.07 | 1 | 0.301 |  |  |  |  |  |  |  |  |  |
|  | E x T x P | **5.66** | **1** | **0.017** |  |  |  |  |  |  |  |  |  |

**Table S8. The evolution of plant temperature-mediated reaction norms.** Output of (generalized) linear mixed models showing effects of temperature-mediated evolution and biotic-mediated evolution on the temperature reaction norms of *Brassica rapa* plant visual and scent traits. Plants of generation one were not included in this analysis. Replicate and cohort were included as random factors in all models. Reaction norms were calculated by comparing traits between half-sibling plants grown at both temperatures. Bold values indicate results where *P* ≤ 0.05. Underlined values indicate results where *P* ≤ 0.07. Scent emission is expressed as pg / flower / l / h. UV relative area calculated by dividing the UV-absorbing surface of the petal (in cm^2^) by the total petal area (in cm^2^). RDR (relative diffuse reflectance) indicates the relative percentage of reflectance.

|  |  | All treatments | | | Bumblebee-genotype | | | Control | | |
| --- | --- | --- | --- | --- | --- | --- | --- | --- | --- | --- |
| Trait | Factor | χ^2^ | DF | P | χ^2^ | DF | P | χ^2^ | DF | P |
| Height | Temperature genotype (T) | 0.32 | 1 | 0.571 | 0.00 | 1 | 0.994 | 0.89 | 1 | 0.344 |
|  | Pollination genotype (P) | 0.84 | 1 | 0.360 |  |  |  |  |  |  |
|  | T x P | 0.48 | 1 | 0.489 |  |  |  |  |  |  |
| Flower number | Temperature genotype (T) | 1.00 | 1 | 0.317 | 1.14 | 1 | 0.286 | 0.00 | 1 | 0.999 |
|  | Pollination genotype (P) | **5.08** | **1** | **0.024** |  |  |  |  |  |  |
|  | T x P | 0.31 | 1 | 0.578 |  |  |  |  |  |  |
| Leaf number | Temperature genotype (T) | 0.33 | 1 | 0.567 | 0.51 | 1 | 0.476 | 0.01 | 1 | 0.937 |
|  | Pollination genotype (P) | 2.54 | 1 | 0.111 |  |  |  |  |  |  |
|  | T x P | 0.19 | 1 | 0.663 |  |  |  |  |  |  |
| Petal area | Temperature genotype (T) | 0.58 | 1 | 0.447 | 1.91 | 1 | 0.167 | 0.18 | 1 | 0.669 |
|  | Pollination genotype (P) | **4.66** | **1** | **0.031** |  |  |  |  |  |  |
|  | T x P | 1.57 | 1 | 0.210 |  |  |  |  |  |  |
| Nectar per plant | Temperature genotype (T) | 3.63 | 1 | 0.057 | **5.24** | **1** | **0.022** | 0.22 | 1 | 0.637 |
|  | Pollination genotype (P) | 1.64 | 1 | 0.201 |  |  |  |  |  |  |
|  | T x P | 2.07 | 1 | 0.150 |  |  |  |  |  |  |
| Nectar per flower | Temperature genotype (T) | 1.62 | 1 | 0.203 | 2.45 | 1 | 0.118 | 0.11 | 1 | 0.743 |
|  | Pollination genotype (P) | 0.17 | 1 | 0.679 |  |  |  |  |  |  |
|  | T x P | 0.83 | 1 | 0.363 |  |  |  |  |  |  |
| Days until flowering | Temperature genotype (T) | 1.62 | 1 | 0.204 | 1.15 | 1 | 0.283 | **4.43** | **1** | **0.035** |
|  | Pollination genotype (P) | 0.04 | 1 | 0.849 |  |  |  |  |  |  |
|  | T x P | **4.51** | **1** | **0.034** |  |  |  |  |  |  |
| UV relative area | Temperature genotype (T) | 0.04 | 1 | 0.849 | 0.93 | 1 | 0.335 | 1.28 | 1 | 0.257 |
|  | Pollination genotype (P) | 0.01 | 1 | 0.907 |  |  |  |  |  |  |
|  | T x P | 2.21 | 1 | 0.137 |  |  |  |  |  |  |
| UVA RDR | Temperature genotype (T) | 0.95 | 1 | 0.330 | 0.38 | 1 | 0.539 | 0.69 | 1 | 0.405 |
|  | Pollination genotype (P) | 0.05 | 1 | 0.829 |  |  |  |  |  |  |
|  | T x P | 0.11 | 1 | 0.739 |  |  |  |  |  |  |
| UVB RDR | Temperature genotype (T) | 0.42 | 1 | 0.519 | 0.00 | 1 | 0.945 | 0.70 | 1 | 0.404 |
|  | Pollination genotype (P) | 0.02 | 1 | 0.879 |  |  |  |  |  |  |
|  | T x P | 0.42 | 1 | 0.516 |  |  |  |  |  |  |
| Yellow | Temperature genotype (T) | 0.42 | 1 | 0.519 | 1.52 | 1 | 0.217 | 0.70 | 1 | 0.403 |
|  | Pollination genotype (P) | 0.02 | 1 | 0.879 |  |  |  |  |  |  |
|  | T x P | 0.42 | 1 | 0.516 |  |  |  |  |  |  |
| Benzaldehyde | Temperature genotype (T) | 0.30 | 1 | 0.582 | 1.01 | 1 | 0.315 | 0.06 | 1 | 0.811 |
|  | Pollination genotype (P) | 0.00 | 1 | 0.985 |  |  |  |  |  |  |
|  | T x P | 0.85 | 1 | 0.356 |  |  |  |  |  |  |
| 1-butene-4-isothyocyanate | Temperature genotype (T) | 0.33 | 1 | 0.566 | 0.50 | 1 | 0.480 | 0.02 | 1 | 0.891 |
|  | Pollination genotype (P) | **3.95** | **1** | **0.047** |  |  |  |  |  |  |
|  | T x P | 0.20 | 1 | 0.656 |  |  |  |  |  |  |
| Methyl benzoate | Temperature genotype (T) | **4.35** | **1** | **0.037** | 0.22 | 1 | 0.639 | **4.99** | **1** | **0.025** |
|  | Pollination genotype (P) | 0.03 | 1 | 0.858 |  |  |  |  |  |  |
|  | T x P | 3.07 | 1 | 0.080 |  |  |  |  |  |  |
| Phenylethyl alcohol | Temperature genotype (T) | 0.35 | 1 | 0.555 | 0.06 | 1 | 0.811 | 1.00 | 1 | 0.316 |
|  | Pollination genotype (P) | 0.01 | 1 | 0.918 |  |  |  |  |  |  |
|  | T x P | 0.79 | 1 | 0.375 |  |  |  |  |  |  |
| 2-Amino benzaldehyde | Temperature genotype (T) | 1.86 | 1 | 0.173 | **9.59** | **1** | **0.002** | 1.26 | 1 | 0.261 |
|  | Pollination genotype (P) | 0.02 | 1 | 0.896 |  |  |  |  |  |  |
|  | T x P | **8.82** | **1** | **0.003** |  |  |  |  |  |  |
| p-Anisaldehyde | Temperature genotype (T) | 1.19 | 1 | 0.275 | 1.76 | 1 | 0.185 | 0.14 | 1 | 0.713 |
|  | Pollination genotype (P) | 0.66 | 1 | 0.417 |  |  |  |  |  |  |
|  | T x P | 0.32 | 1 | 0.574 |  |  |  |  |  |  |
| Methyl anthranilate | Temperature genotype (T) | 1.33 | 1 | 0.249 | **7.21** | **1** | **0.007** | 0.67 | 1 | 0.412 |
|  | Pollination genotype (P) | 0.13 | 1 | 0.713 |  |  |  |  |  |  |
|  | T x P | **5.70** | **1** | **0.017** |  |  |  |  |  |  |
| *(Z)*-3-Hexen-1-ol acetate | Temperature genotype (T) | 0.71 | 1 | 0.398 | 0.01 | 1 | 0.926 | 1.50 | 1 | 0.220 |
|  | Pollination genotype (P) | 1.11 | 1 | 0.291 |  |  |  |  |  |  |
|  | T x P | 0.95 | 1 | 0.330 |  |  |  |  |  |  |
| Phenylacetaldehyde | Temperature genotype (T) | 0.00 | 1 | 0.948 | 0.58 | 1 | 0.448 | 0.29 | 1 | 0.588 |
|  | Pollination genotype (P) | 1.52 | 1 | 0.217 |  |  |  |  |  |  |
|  | T x P | 0.82 | 1 | 0.366 |  |  |  |  |  |  |
| Benzyl nitrile | Temperature genotype (T) | 2.81 | 1 | 0.094 | 2.37 | 1 | 0.123 | 0.01 | 1 | 0.922 |
|  | Pollination genotype (P) | 0.04 | 1 | 0.833 |  |  |  |  |  |  |
|  | T x P | **7.36** | **1** | **0.007** |  |  |  |  |  |  |
| Methyl salicilate | Temperature genotype (T) | 2.81 | 1 | 0.094 | 0.51 | 1 | 0.477 | **10.41** | **1** | **0.001** |
|  | Pollination genotype (P) | 0.04 | 1 | 0.833 |  |  |  |  |  |  |
|  | T x P | **7.36** | **1** | **0.007** |  |  |  |  |  |  |
| Indole | Temperature genotype (T) | 3.09 | 1 | 0.079 | **5.20** | **1** | **0.023** | 0.04 | 1 | 0.839 |
|  | Pollination genotype (P) | 0.12 | 1 | 0.726 |  |  |  |  |  |  |
|  | T x P | 2.17 | 1 | 0.141 |  |  |  |  |  |  |
| (E,E)-α-Farnesene | Temperature genotype (T) | 0.47 | 1 | 0.494 | **5.54** | **1** | **0.019** | 3.09 | 1 | 0.079 |
|  | Pollination genotype (P) | 0.80 | 1 | 0.371 |  |  |  |  |  |  |
|  | T x P | **8.63** | **1** | **0.003** |  |  |  |  |  |  |
| Total volatile emission | Temperature genotype (T) | 1.20 | 1 | 0.274 | **7.21** | **1** | **0.007** | 1.86 | 1 | 0.173 |
|  | Pollination genotype (P) | 0.16 | 1 | 0.690 |  |  |  |  |  |  |
|  | T x P | **8.49** | **1** | **0.004** |  |  |  |  |  |  |

**Table S9. The effects of temperature- and pollinator-mediated evolution on plant temperature reaction norms.** Average reaction norm values for plant sibling pairs evolved at two temperatures, pollination genotype treatments, and grown at local and foreign temperatures. Means ± S.E. of the temperature reaction norms of *Brassica rapa* half-sibling pair plant traits. Reaction norms were calculated by comparing traits between half-sibling plants grown at both temperatures.

|  |  | Control | | Bumblebee-genotype | |
| --- | --- | --- | --- | --- | --- |
|  | number | Amb-genotype | Hot-genotype | Amb-genotype | Hot-genotype |
| Height | 275 | 2.59 ± 0.89 | 3.99 ± 1.14 | 4.36 ± 1.20 | 4.38 ± 1.09 |
|  |  |  |  |  |  |
| Leaf Number | 275 | 1.90 ± 0.40 | 1.84 ± 0.40 | 2.75 ± 0.41 | 2.31 ± 0.47 |
|  |  |  |  |  |  |
| Flower number | 275 | 4.10 ± 1.44 | 4.06 ± 1.15 | 5.77 ± 1.42 | 7.87 ± 1.60 |
|  |  |  |  |  |  |
| Nectar | 236 | 0 ± 0.008 | 0.003 ± 0.007 | -0.003 ± 0.007 | 0.013 ± 0.008 |
|  |  |  |  |  |  |
| Nectar per plant | 180 | 0.35 ± 0.25 | 0.49 ± 0.14 | 0.31 ± 0.23 | 1.13 ± 0.28 |
|  |  |  |  |  |  |
| Days to flower | 278 | -1.96 ± 0.19 | -1.46 ± 0.13 | -1.54 ± 0.15 | -1.75 ± 0.13 |
|  |  |  |  |  |  |
| petal area | 239 | -0.07 ± 0.01 | -0.06 ± 0.01 | -0.04 ± 0.01 | -0.06 ± 0.01 |
|  |  |  |  |  |  |
| UV relative area | 239 | -0.02 ± 0.02 | 0.01 ± 0.02 | 0.01 ± 0.02 | -0.01 ± 0.02 |
|  |  |  |  |  |  |
| UVA RDR | 238 | 2.55 ± 2.39 | -0.85 ± 3.28 | 1.13 ± 2.33 | -0.54 ± 2.25 |
|  |  |  |  |  |  |
| UVB RDR | 238 | 1.92 ± 2.52 | -1.23 ± 2.76 | -0.02 ± 2.21 | 0.01 ± 2.15 |
|  |  |  |  |  |  |
| Yellow | 238 | -0.58 ± 1.48 | 1.20 ± 1.56 | 2.49 ± 1.23 | 0.41 ± 1.16 |
|  |  |  |  |  |  |
| Benzaldehyde | 236 | -0.08 ± 0.08 | -0.06 ± 0.07 | -0.02 ± 0.07 | -0.12 ± 0.07 |
|  |  |  |  |  |  |
| 1-Butene-4-isothyocyanate | 236 | 0.48 ± 0.08 | 0.47 ± 0.08 | 0.37 ± 0.07 | 0.29 ± 0.08 |
|  |  |  |  |  |  |
| Methyl Benzoate | 236 | -0.11 ± 0.04 | 0.02 ± 0.04 | -0.05 ± 0.02 | -0.03 ± 0.02 |
|  |  |  |  |  |  |
| Phenylethyl alcohol | 236 | -0.01 ± 0.01 | -0.03 ± 0.01 | -0.02 ± 0.01 | -0.02 ± 0.01 |
|  |  |  |  |  |  |
| 2-Amino benzaldehyde | 236 | -0.51 ± 0.14 | -0.28 ± 0.15 | -0.1 ± 0.14 | -0.72 ± 0.14 |
|  |  |  |  |  |  |
| p-Anisaldehyde | 236 | -0.04 ± 0.02 | -0.03 ± 0.01 | -0.06 ± 0.02 | -0.03 ± 0.01 |
|  |  |  |  |  |  |
| Methyl anthranilate | 236 | -0.22 ± 0.07 | -0.14 ± 0.08 | -0.03 ± 0.06 | -0.28 ± 0.07 |
|  |  |  |  |  |  |
| *(Z)*-3-Hexen-1-ol acetate | 236 | 0.11 ± 0.06 | 0.01 ± 0.05 | 0.11 ± 0.05 | 0.13 ± 0.05 |
|  |  |  |  |  |  |
| Phenylacetaldehyde | 236 | -0.10 ± 0.03 | -0.12 ± 0.03 | -0.09 ± 0.03 | -0.06 ± 0.01 |
|  |  |  |  |  |  |
| Benzyl Nitrile | 236 | -0.31 ± 0.05 | -0.30 ± 0.06 | -0.21 ± 0.05 | -0.31 ± 0.05 |
|  |  |  |  |  |  |
| Methyl salicilate | 236 | -0.10 ± 0.02 | 0.00 ± 0.02 | -0.05 ± 0.03 | -0.07 ± 0.02 |
|  |  |  |  |  |  |
| Indole | 236 | -0.23 ± 0.08 | -0.26 ± 0.10 | -0.13 ± 0.10 | -0.42 ± 0.08 |
|  |  |  |  |  |  |
| *(E,E)*-α-Farnesene | 236 | -0.5 ± 0.10 | -0.24 ± 0.11 | -0.26 ± 0.13 | -0.67 ± 0.11 |
|  |  |  |  |  |  |
| Total volatile emission | 236 | -0.4 ± 0.09 | -0.21 ± 0.11 | -0.13 ± 0.12 | -0.55 ± 0.11 |

**Table S10. Plastic and evolutionary changes in honest signaling**. Output of (generalized) linear mixed models showing effects of temperature environment, temperature-mediated evolution and biotic-mediated evolution on the honest signaling (correlation with nectar per flower-µl-) of *Brassica rapa* plant traits. Replicate and cohort were included in all models as random factors. Bold values indicate results where *P* ≤ 0.05. Underlined values indicate results where *P* ≤ 0.07. Scent emission is expressed as pg / flower / l / h. UV relative area calculated by dividing the UV-absorbing surface of the petal (in cm^2^) by the total petal area (in cm^2^). RDR (relative diffuse reflectance) indicates the relative percentage of reflectance.

|  |  | All treatments | | | Bumblebee-genotype | | | Control | | |
| --- | --- | --- | --- | --- | --- | --- | --- | --- | --- | --- |
| Trait | Factor | χ^2^ | DF | P | χ^2^ | DF | P | χ^2^ | DF | P |
| Height | Trait (A) | **21.99** | **1** | **<0.001** | **17.21** | **1** | **<0.001** | **5.39** | **1** | **0.020** |
|  | Temperature environment (E) | 0.25 | 1 | 0.616 | 0.45 | 1 | 0.501 | 0.00 | 1 | 0.956 |
|  | Temperature genotype (T) | 0.02 | 1 | 0.889 | **3.89** | **1** | **0.048** | 2.92 | 1 | 0.087 |
|  | Pollination genotype (P) | **18.94** | **1** | **<0.001** |  |  |  |  |  |  |
|  | A x E | 1.51 | 1 | 0.218 | 3.28 | 1 | 0.070 | 0.01 | 1 | 0.930 |
|  | A x T | 0.90 | 1 | 0.343 | 0.00 | 1 | 0.956 | 1.44 | 1 | 0.230 |
|  | E x T | 0.73 | 1 | 0.394 | 2.70 | 1 | 0.101 | 0.07 | 1 | 0.789 |
|  | A x P | **4.18** | **1** | **0.041** |  |  |  |  |  |  |
|  | E x P | 0.44 | 1 | 0.509 |  |  |  |  |  |  |
|  | T x P | **6.77** | **1** | **0.009** |  |  |  |  |  |  |
|  | A x E x T | 0.07 | 1 | 0.789 | 1.52 | 1 | 0.218 | 0.62 | 1 | 0.430 |
|  | A x E x P | 1.92 | 1 | 0.166 |  |  |  |  |  |  |
|  | A x T x P | 0.63 | 1 | 0.429 |  |  |  |  |  |  |
|  | E x T x P | 1.96 | 1 | 0.162 |  |  |  |  |  |  |
|  | A x E x T x P | 1.61 | 1 | 0.205 |  |  |  |  |  |  |
| Leaf number | Trait (A) | **20.13** | **1** | **<0.001** | **13.05** | **1** | **<0.001** | **6.97** | **1** | **0.008** |
|  | Temperature environment (E) | 0.31 | 1 | 0.579 | 0.49 | 1 | 0.483 | 0.02 | 1 | 0.888 |
|  | Temperature genotype (T) | 0.29 | 1 | 0.588 | 0.54 | 1 | 0.464 | 2.20 | 1 | 0.138 |
|  | Pollination genotype (P) | **21.91** | **1** | **<0.001** |  |  |  |  |  |  |
|  | A x E | 0.46 | 1 | 0.496 | 0.04 | 1 | 0.836 | 0.36 | 1 | 0.550 |
|  | A x T | 1.03 | 1 | 0.310 | 0.00 | 1 | 0.953 | 1.62 | 1 | 0.203 |
|  | E x T | 0.67 | 1 | 0.412 | 1.77 | 1 | 0.183 | 0.00 | 1 | 0.976 |
|  | A x P | 0.78 | 1 | 0.377 |  |  |  |  |  |  |
|  | E x P | 0.27 | 1 | 0.602 |  |  |  |  |  |  |
|  | T x P | 2.24 | 1 | 0.134 |  |  |  |  |  |  |
|  | A x E x T | 0.70 | 1 | 0.402 | **5.07** | **1** | **0.024** | 0.39 | 1 | 0.533 |
|  | A x E x P | 0.08 | 1 | 0.774 |  |  |  |  |  |  |
|  | A x T x P | 0.55 | 1 | 0.460 |  |  |  |  |  |  |
|  | E x T x P | 0.97 | 1 | 0.324 |  |  |  |  |  |  |
|  | A x E x T x P | **3.90** | **1** | **0.048** |  |  |  |  |  |  |
| Flower number | Trait (A) | 6.18 | 1 | **0.013** | **5.43** | **1** | **0.020** | 0.58 | 1 | 0.448 |
|  | Temperature environment (E) | 0.09 | 1 | 0.759 | 0.00 | 1 | 0.997 | 0.21 | 1 | 0.648 |
|  | Temperature genotype (T) | 0.04 | 1 | 0.833 | 1.77 | 1 | 0.183 | 2.57 | 1 | 0.109 |
|  | Pollination genotype (P) | **22.45** | **1** | **<0.001** |  |  |  |  |  |  |
|  | A x E | 0.19 | 1 | 0.664 | 0.09 | 1 | 0.759 | 0.09 | 1 | 0.769 |
|  | A x T | 0.69 | 1 | 0.407 | 1.19 | 1 | 0.275 | 0.11 | 1 | 0.735 |
|  | E x T | 1.34 | 1 | 0.247 | 2.24 | 1 | 0.134 | 0.08 | 1 | 0.779 |
|  | A x P | 1.69 | 1 | 0.194 |  |  |  |  |  |  |
|  | E x P | 0.11 | 1 | 0.741 |  |  |  |  |  |  |
|  | T x P | **5.13** | **1** | **0.024** |  |  |  |  |  |  |
|  | A x E x T | 2.37 | 1 | 0.124 | 1.02 | 1 | 0.313 | 1.44 | 1 | 0.230 |
|  | A x E x P | 0.04 | 1 | 0.844 |  |  |  |  |  |  |
|  | A x T x P | 0.37 | 1 | 0.545 |  |  |  |  |  |  |
|  | E x T x P | 0.38 | 1 | 0.535 |  |  |  |  |  |  |
|  | A x E x T x P | 0.03 | 1 | 0.867 |  |  |  |  |  |  |
| Petal area | Trait (A) | **36.13** | **1** | **<0.001** | **26.88** | **1** | **<0.001** | **12.82** | **1** | **<0.001** |
|  | Temperature environment (E) | **20.22** | **1** | **<0.001** | **13.83** | **1** | **<0.001** | **7.55** | **1** | **0.006** |
|  | Temperature genotype (T) | 0.04 | 1 | 0.834 | 3.10 | 1 | 0.078 | **3.99** | **1** | **0.046** |
|  | Pollination genotype (P) | **9.33** | **1** | **0.002** |  |  |  |  |  |  |
|  | A x E | 0.00 | 1 | 0.949 | 2.36 | 1 | 0.124 | 2.41 | 1 | 0.121 |
|  | A x T | 2.49 | 1 | 0.114 | 2.13 | 1 | 0.145 | 0.16 | 1 | 0.692 |
|  | E x T | **4.90** | **1** | **0.027** | **6.14** | **1** | **0.013** | 0.17 | 1 | 0.681 |
|  | A x P | 3.37 | 1 | 0.066 |  |  |  |  |  |  |
|  | E x P | 1.00 | 1 | 0.317 |  |  |  |  |  |  |
|  | T x P | **7.59** | **1** | **0.006** |  |  |  |  |  |  |
|  | A x E x T | 1.78 | 1 | 0.183 | 2.47 | 1 | 0.116 | 0.56 | 1 | 0.455 |
|  | A x E x P | **4.79** | **1** | **0.029** |  |  |  |  |  |  |
|  | A x T x P | 0.98 | 1 | 0.323 |  |  |  |  |  |  |
|  | E x T x P | 2.63 | 1 | 0.105 |  |  |  |  |  |  |
|  | A x E x T x P | 0.09 | 1 | 0.763 |  |  |  |  |  |  |
| UV relative area | Trait (A) | 0.72 | 1 | 0.397 | 3.13 | 1 | 0.077 | 0.14 | 1 | 0.713 |
|  | Temperature environment (E) | 1.37 | 1 | 0.243 | 0.82 | 1 | 0.364 | 0.46 | 1 | 0.498 |
|  | Temperature genotype (T) | 0.02 | 1 | 0.875 | 2.49 | 1 | 0.114 | 2.94 | 1 | 0.087 |
|  | Pollination genotype (P) | **17.74** | **1** | **<0.001** |  |  |  |  |  |  |
|  | A x E | 1.22 | 1 | 0.268 | 2.07 | 1 | 0.150 | 0.01 | 1 | 0.933 |
|  | A x T | 0.01 | 1 | 0.916 | 0.35 | 1 | 0.554 | 0.80 | 1 | 0.372 |
|  | E x T | 1.66 | 1 | 0.197 | 2.62 | 1 | 0.105 | 0.06 | 1 | 0.809 |
|  | A x P | 2.61 | 1 | 0.106 |  |  |  |  |  |  |
|  | E x P | 0.19 | 1 | 0.660 |  |  |  |  |  |  |
|  | T x P | **5.07** | **1** | **0.024** |  |  |  |  |  |  |
|  | A x E x T | 2.14 | 1 | 0.143 | **4.50** | **1** | **0.034** | 0.12 | 1 | 0.734 |
|  | A x E x P | 1.56 | 1 | 0.211 |  |  |  |  |  |  |
|  | A x T x P | 1.44 | 1 | 0.230 |  |  |  |  |  |  |
|  | E x T x P | 1.65 | 1 | 0.198 |  |  |  |  |  |  |
|  | A x E x T x P | 2.54 | 1 | 0.111 |  |  |  |  |  |  |
| UVA RDR | Trait (A) | 0.35 | 1 | 0.556 | 0.28 | 1 | 0.599 | 1.08 | 1 | 0.298 |
|  | Temperature environment (E) | 1.19 | 1 | 0.276 | 0.80 | 1 | 0.370 | 0.35 | 1 | 0.552 |
|  | Temperature genotype (T) | 0.03 | 1 | 0.869 | 1.68 | 1 | 0.195 | 2.23 | 1 | 0.136 |
|  | Pollination genotype (P) | **19.67** | **1** | **<0.001** |  |  |  |  |  |  |
|  | A x E | 0.01 | 1 | 0.905 | 0.03 | 1 | 0.868 | 0.02 | 1 | 0.882 |
|  | A x T | 0.00 | 1 | 0.962 | 0.01 | 1 | 0.925 | 0.00 | 1 | 0.977 |
|  | E x T | 1.46 | 1 | 0.227 | 1.78 | 1 | 0.182 | 0.12 | 1 | 0.725 |
|  | A x P | 0.72 | 1 | 0.396 |  |  |  |  |  |  |
|  | E x P | 0.05 | 1 | 0.830 |  |  |  |  |  |  |
|  | T x P | **3.88** | **1** | **0.049** |  |  |  |  |  |  |
|  | A x E x T | 0.02 | 1 | 0.901 | 0.03 | 1 | 0.855 | 0.09 | 1 | 0.770 |
|  | A x E x P | 0.07 | 1 | 0.787 |  |  |  |  |  |  |
|  | A x T x P | 0.02 | 1 | 0.881 |  |  |  |  |  |  |
|  | E x T x P | 0.60 | 1 | 0.440 |  |  |  |  |  |  |
|  | A x E x T x P | 0.00 | 1 | 0.968 |  |  |  |  |  |  |
| UVB RDR | Trait (A) | 0.53 | 1 | 0.465 | 0.25 | 1 | 0.614 | 0.27 | 1 | 0.607 |
|  | Temperature environment (E) | 1.30 | 1 | 0.254 | 0.83 | 1 | 0.362 | 0.42 | 1 | 0.515 |
|  | Temperature genotype (T) | 0.18 | 1 | 0.672 | 1.40 | 1 | 0.236 | 2.99 | 1 | 0.084 |
|  | Pollination genotype (P) | **19.23** | **1** | **<0.001** |  |  |  |  |  |  |
|  | A x E | 1.25 | 1 | 0.264 | 1.24 | 1 | 0.266 | 0.24 | 1 | 0.627 |
|  | A x T | 0.54 | 1 | 0.461 | 2.16 | 1 | 0.141 | 0.31 | 1 | 0.576 |
|  | E x T | 1.38 | 1 | 0.240 | 2.00 | 1 | 0.157 | 0.08 | 1 | 0.775 |
|  | A x P | 0.01 | 1 | 0.910 |  |  |  |  |  |  |
|  | E x P | 0.08 | 1 | 0.772 |  |  |  |  |  |  |
|  | T x P | **4.52** | **1** | **0.034** |  |  |  |  |  |  |
|  | A x E x T | 0.03 | 1 | 0.859 | 0.18 | 1 | 0.669 | 0.11 | 1 | 0.737 |
|  | A x E x P | 0.27 | 1 | 0.601 |  |  |  |  |  |  |
|  | A x T x P | 3.18 | 1 | 0.075 |  |  |  |  |  |  |
|  | E x T x P | 0.77 | 1 | 0.379 |  |  |  |  |  |  |
|  | A x E x T x P | 0.27 | 1 | 0.606 |  |  |  |  |  |  |
| Yellow RDR | Trait (A) | 0.01 | 1 | 0.931 | 0.43 | 1 | 0.514 | 0.45 | 1 | 0.504 |
|  | Temperature environment (E) | 1.20 | 1 | 0.273 | 0.82 | 1 | 0.365 | 0.40 | 1 | 0.529 |
|  | Temperature genotype (T) | 0.12 | 1 | 0.731 | 1.58 | 1 | 0.209 | 2.82 | 1 | 0.093 |
|  | Pollination genotype (P) | **18.61** | **1** | **<0.001** |  |  |  |  |  |  |
|  | A x E | 0.02 | 1 | 0.877 | 0.01 | 1 | 0.930 | 0.00 | 1 | 0.946 |
|  | A x T | 0.82 | 1 | 0.364 | 0.64 | 1 | 0.423 | 0.17 | 1 | 0.679 |
|  | E x T | 1.35 | 1 | 0.246 | 1.91 | 1 | 0.167 | 0.07 | 1 | 0.788 |
|  | A x P | 0.22 | 1 | 0.637 |  |  |  |  |  |  |
|  | E x P | 0.04 | 1 | 0.850 |  |  |  |  |  |  |
|  | T x P | **4.64** | **1** | **0.031** |  |  |  |  |  |  |
|  | A x E x T | 0.08 | 1 | 0.772 | 0.80 | 1 | 0.371 | 0.29 | 1 | 0.591 |
|  | A x E x P | 0.00 | 1 | 0.966 |  |  |  |  |  |  |
|  | A x T x P | 0.31 | 1 | 0.576 |  |  |  |  |  |  |
|  | E x T x P | 0.79 | 1 | 0.375 |  |  |  |  |  |  |
|  | A x E x T x P | 1.36 | 1 | 0.243 |  |  |  |  |  |  |
| Benzaldehyde | Trait (A) | 0.17 | 1 | 0.682 | 0.00 | 1 | 0.947 | 0.38 | 1 | 0.539 |
|  | Temperature environment (E) | 0.71 | 1 | 0.399 | 0.59 | 1 | 0.442 | 0.16 | 1 | 0.689 |
|  | Temperature genotype (T) | 0.05 | 1 | 0.818 | 1.28 | 1 | 0.257 | 2.11 | 1 | 0.146 |
|  | Pollination genotype (P) | **16.09** | **1** | **<0.001** |  |  |  |  |  |  |
|  | A x E | 3.85 | 1 | 0.050 | 0.21 | 1 | 0.645 | **4.40** | **1** | **0.036** |
|  | A x T | 0.66 | 1 | 0.415 | 1.94 | 1 | 0.164 | 0.18 | 1 | 0.670 |
|  | E x T | 2.05 | 1 | 0.152 | 2.06 | 1 | 0.151 | 0.34 | 1 | 0.561 |
|  | A x P | 1.35 | 1 | 0.245 |  |  |  |  |  |  |
|  | E x P | 0.02 | 1 | 0.881 |  |  |  |  |  |  |
|  | T x P | 3.04 | 1 | 0.081 |  |  |  |  |  |  |
|  | A x E x T | 0.91 | 1 | 0.341 | **4.90** | **1** | **0.027** | 0.57 | 1 | 0.449 |
|  | A x E x P | 0.61 | 1 | 0.436 |  |  |  |  |  |  |
|  | A x T x P | 2.69 | 1 | 0.101 |  |  |  |  |  |  |
|  | E x T x P | 0.63 | 1 | 0.426 |  |  |  |  |  |  |
|  | A x E x T x P | **4.93** | **1** | **0.026** |  |  |  |  |  |  |
| 1-butene-4-isothyocyanate | Trait (A) | 0.01 | 1 | 0.916 | 0.32 | 1 | 0.573 | 0.03 | 1 | 0.867 |
|  | Temperature environment (E) | 0.77 | 1 | 0.381 | 0.38 | 1 | 0.540 | 0.24 | 1 | 0.627 |
|  | Temperature genotype (T) | 0.00 | 1 | 0.989 | 1.45 | 1 | 0.229 | 1.80 | 1 | 0.180 |
|  | Pollination genotype (P) | **16.06** | **1** | **<0.001** |  |  |  |  |  |  |
|  | A x E | 0.32 | 1 | 0.572 | 1.13 | 1 | 0.287 | 0.00 | 1 | 0.999 |
|  | A x T | 0.00 | 1 | 0.965 | 0.01 | 1 | 0.939 | 0.00 | 1 | 0.999 |
|  | E x T | 2.08 | 1 | 0.150 | 2.30 | 1 | 0.129 | 0.24 | 1 | 0.625 |
|  | A x P | 0.96 | 1 | 0.328 |  |  |  |  |  |  |
|  | E x P | 0.01 | 1 | 0.931 |  |  |  |  |  |  |
|  | T x P | 3.10 | 1 | 0.078 |  |  |  |  |  |  |
|  | A x E x T | 0.07 | 1 | 0.786 | 0.46 | 1 | 0.500 | 0.21 | 1 | 0.644 |
|  | A x E x P | 0.42 | 1 | 0.515 |  |  |  |  |  |  |
|  | A x T x P | 0.00 | 1 | 0.960 |  |  |  |  |  |  |
|  | E x T x P | 0.58 | 1 | 0.448 |  |  |  |  |  |  |
|  | A x E x T x P | 0.61 | 1 | 0.435 |  |  |  |  |  |  |
| Methyl benzoate | Trait (A) | **9.80** | **1** | **0.002** | **9.08** | **1** | **0.003** | 2.72 | 1 | 0.099 |
|  | Temperature environment (E) | 1.76 | 1 | 0.185 | 1.33 | 1 | 0.248 | 0.47 | 1 | 0.495 |
|  | Temperature genotype (T) | 0.01 | 1 | 0.924 | 1.80 | 1 | 0.180 | 1.40 | 1 | 0.237 |
|  | Pollination genotype (P) | **8.79** | **1** | **0.003** |  |  |  |  |  |  |
|  | A x E | 0.89 | 1 | 0.344 | 0.11 | 1 | 0.736 | 1.98 | 1 | 0.159 |
|  | A x T | 0.46 | 1 | 0.496 | 0.98 | 1 | 0.322 | 0.05 | 1 | 0.825 |
|  | E x T | 1.82 | 1 | 0.177 | 2.39 | 1 | 0.122 | 0.15 | 1 | 0.702 |
|  | A x P | **5.11** | **1** | **0.024** |  |  |  |  |  |  |
|  | E x P | 0.02 | 1 | 0.880 |  |  |  |  |  |  |
|  | T x P | 3.68 | 1 | 0.055 |  |  |  |  |  |  |
|  | A x E x T | 0.00 | 1 | 0.993 | 1.84 | 1 | 0.174 | 0.62 | 1 | 0.432 |
|  | A x E x P | 1.29 | 1 | 0.256 |  |  |  |  |  |  |
|  | A x T x P | 0.53 | 1 | 0.467 |  |  |  |  |  |  |
|  | E x T x P | 0.79 | 1 | 0.375 |  |  |  |  |  |  |
|  | A x E x T x P | 2.50 | 1 | 0.114 |  |  |  |  |  |  |
| Phenylethyl alcohol | Trait (A) | **14.14** | **1** | **<0.001** | **15.51** | **1** | **<0.001** | 0.45 | 1 | 0.504 |
|  | Temperature environment (E) | 2.16 | 1 | 0.142 | 2.14 | 1 | 0.143 | 0.33 | 1 | 0.564 |
|  | Temperature genotype (T) | 0.00 | 1 | 0.998 | 2.02 | 1 | 0.155 | 1.88 | 1 | 0.171 |
|  | Pollination genotype (P) | **15.43** | **1** | **<0.001** |  |  |  |  |  |  |
|  | A x E | 2.75 | 1 | 0.097 | 1.76 | 1 | 0.184 | 1.15 | 1 | 0.284 |
|  | A x T | 0.09 | 1 | 0.767 | 1.31 | 1 | 0.253 | 1.18 | 1 | 0.277 |
|  | E x T | 1.69 | 1 | 0.193 | 0.99 | 1 | 0.319 | 0.68 | 1 | 0.409 |
|  | A x P | **6.30** | **1** | **0.012** |  |  |  |  |  |  |
|  | E x P | 0.79 | 1 | 0.376 |  |  |  |  |  |  |
|  | T x P | **3.99** | **1** | **0.046** |  |  |  |  |  |  |
|  | A x E x T | 0.50 | 1 | 0.481 | 0.24 | 1 | 0.626 | 0.84 | 1 | 0.360 |
|  | A x E x P | 0.67 | 1 | 0.413 |  |  |  |  |  |  |
|  | A x T x P | 2.19 | 1 | 0.139 |  |  |  |  |  |  |
|  | E x T x P | 0.03 | 1 | 0.861 |  |  |  |  |  |  |
|  | A x E x T x P | 0.75 | 1 | 0.386 |  |  |  |  |  |  |
| 2-Amino benzaldehyde | Trait (A) | **12.36** | **1** | **<0.001** | **9.97** | **1** | **0.002** | 2.54 | 1 | 0.111 |
|  | Temperature environment (E) | 2.40 | 1 | 0.122 | 1.95 | 1 | 0.163 | 0.49 | 1 | 0.485 |
|  | Temperature genotype (T) | 0.21 | 1 | 0.650 | 0.92 | 1 | 0.338 | 2.54 | 1 | 0.111 |
|  | Pollination genotype (P) | **11.38** | **1** | **0.001** |  |  |  |  |  |  |
|  | A x E | 1.22 | 1 | 0.270 | 2.12 | 1 | 0.145 | 0.07 | 1 | 0.791 |
|  | A x T | 0.89 | 1 | 0.346 | 0.13 | 1 | 0.718 | 1.95 | 1 | 0.163 |
|  | E x T | 3.77 | 1 | 0.052 | 3.64 | 1 | 0.057 | 0.57 | 1 | 0.448 |
|  | A x P | 3.32 | 1 | 0.068 |  |  |  |  |  |  |
|  | E x P | 0.65 | 1 | 0.420 |  |  |  |  |  |  |
|  | T x P | 3.42 | 1 | 0.064 |  |  |  |  |  |  |
|  | A x E x T | **4.83** | **1** | **0.028** | **6.18** | **1** | **0.013** | 0.56 | 1 | 0.455 |
|  | A x E x P | 0.97 | 1 | 0.325 |  |  |  |  |  |  |
|  | A x T x P | 1.65 | 1 | 0.199 |  |  |  |  |  |  |
|  | E x T x P | 1.28 | 1 | 0.257 |  |  |  |  |  |  |
|  | A x E x T x P | 2.47 | 1 | 0.116 |  |  |  |  |  |  |
| p-Anisaldehyde | Trait (A) | 2.37 | 1 | 0.124 | 2.09 | 1 | 0.148 | 0.14 | 1 | 0.713 |
|  | Temperature environment (E) | 1.46 | 1 | 0.227 | 1.28 | 1 | 0.259 | 0.27 | 1 | 0.606 |
|  | Temperature genotype (T) | 0.04 | 1 | 0.833 | 2.80 | 1 | 0.094 | 2.04 | 1 | 0.154 |
|  | Pollination genotype (P) | **14.66** | **1** | **<0.001** |  |  |  |  |  |  |
|  | A x E | 0.03 | 1 | 0.874 | 0.00 | 1 | 0.978 | 0.10 | 1 | 0.758 |
|  | A x T | 1.20 | 1 | 0.274 | 0.11 | 1 | 0.739 | 1.06 | 1 | 0.304 |
|  | E x T | 2.16 | 1 | 0.141 | 1.94 | 1 | 0.163 | 0.50 | 1 | 0.482 |
|  | A x P | 2.85 | 1 | 0.091 |  |  |  |  |  |  |
|  | E x P | 0.61 | 1 | 0.436 |  |  |  |  |  |  |
|  | T x P | **5.68** | **1** | **0.017** |  |  |  |  |  |  |
|  | A x E x T | 1.19 | 1 | 0.275 | 2.08 | 1 | 0.149 | 0.04 | 1 | 0.848 |
|  | A x E x P | 0.03 | 1 | 0.862 |  |  |  |  |  |  |
|  | A x T x P | 0.07 | 1 | 0.785 |  |  |  |  |  |  |
|  | E x T x P | 0.42 | 1 | 0.518 |  |  |  |  |  |  |
|  | A x E x T x P | 1.55 | 1 | 0.213 |  |  |  |  |  |  |
| Methyl anthranilate | Trait (A) | 3.74 | 1 | 0.053 | **6.66** | **1** | **0.010** | 0.03 | 1 | 0.868 |
|  | Temperature environment (E) | 1.39 | 1 | 0.238 | 1.51 | 1 | 0.219 | 0.18 | 1 | 0.672 |
|  | Temperature genotype (T) | 0.24 | 1 | 0.623 | 0.62 | 1 | 0.431 | 2.03 | 1 | 0.155 |
|  | Pollination genotype (P) | **14.26** | **1** | **<0.001** |  |  |  |  |  |  |
|  | A x E | 0.52 | 1 | 0.472 | 0.47 | 1 | 0.491 | 0.13 | 1 | 0.719 |
|  | A x T | 1.00 | 1 | 0.318 | 0.10 | 1 | 0.749 | 2.22 | 1 | 0.136 |
|  | E x T | 3.10 | 1 | 0.078 | 2.75 | 1 | 0.097 | 0.66 | 1 | 0.417 |
|  | A x P | **5.53** | **1** | **0.019** |  |  |  |  |  |  |
|  | E x P | 0.41 | 1 | 0.524 |  |  |  |  |  |  |
|  | T x P | 2.50 | 1 | 0.114 |  |  |  |  |  |  |
|  | A x E x T | 1.82 | 1 | 0.177 | 3.01 | 1 | 0.083 | 0.14 | 1 | 0.708 |
|  | A x E x P | 0.08 | 1 | 0.772 |  |  |  |  |  |  |
|  | A x T x P | 1.23 | 1 | 0.267 |  |  |  |  |  |  |
|  | E x T x P | 0.72 | 1 | 0.396 |  |  |  |  |  |  |
|  | A x E x T x P | 1.42 | 1 | 0.234 |  |  |  |  |  |  |
| *(Z)*-3-Hexen-1-ol acetate | Trait (A) | 0.02 | 1 | 0.878 | 0.12 | 1 | 0.731 | 0.21 | 1 | 0.647 |
|  | Temperature environment (E) | 0.75 | 1 | 0.387 | 0.50 | 1 | 0.480 | 0.26 | 1 | 0.613 |
|  | Temperature genotype (T) | 0.11 | 1 | 0.740 | 0.99 | 1 | 0.319 | 2.00 | 1 | 0.157 |
|  | Pollination genotype (P) | **16.32** | **1** | **<0.001** |  |  |  |  |  |  |
|  | A x E | 0.01 | 1 | 0.926 | 0.08 | 1 | 0.778 | 0.00 | 1 | 0.972 |
|  | A x T | 3.56 | 1 | 0.059 | **10.73** | **1** | **<0.001** | 0.06 | 1 | 0.805 |
|  | E x T | 0.84 | 1 | 0.359 | 0.63 | 1 | 0.429 | 0.30 | 1 | 0.586 |
|  | A x P | 0.17 | 1 | 0.681 |  |  |  |  |  |  |
|  | E x P | 0.04 | 1 | 0.851 |  |  |  |  |  |  |
|  | T x P | **4.23** | **1** | **0.040** |  |  |  |  |  |  |
|  | A x E x T | 0.24 | 1 | 0.622 | 0.37 | 1 | 0.541 | 1.31 | 1 | 0.252 |
|  | A x E x P | 0.06 | 1 | 0.804 |  |  |  |  |  |  |
|  | A x T x P | **8.58** | **1** | **0.003** |  |  |  |  |  |  |
|  | E x T x P | 0.02 | 1 | 0.898 |  |  |  |  |  |  |
|  | A x E x T x P | 1.61 | 1 | 0.205 |  |  |  |  |  |  |
| Phenylacetaldehyde | Trait (A) | **11.70** | **1** | **0.001** | **15.18** | **1** | **<0.001** | 0.10 | 1 | 0.757 |
|  | Temperature environment (E) | 2.44 | 1 | 0.118 | 2.66 | 1 | 0.103 | 0.27 | 1 | 0.604 |
|  | Temperature genotype (T) | 0.02 | 1 | 0.888 | 2.61 | 1 | 0.106 | 1.94 | 1 | 0.163 |
|  | Pollination genotype (P) | **14.80** | **1** | **<0.001** |  |  |  |  |  |  |
|  | A x E | 1.49 | 1 | 0.222 | 0.89 | 1 | 0.346 | 0.58 | 1 | 0.447 |
|  | A x T | 1.21 | 1 | 0.271 | 0.04 | 1 | 0.840 | 1.71 | 1 | 0.190 |
|  | E x T | 2.42 | 1 | 0.120 | 1.36 | 1 | 0.244 | 0.97 | 1 | 0.326 |
|  | A x P | **9.51** | **1** | **0.002** |  |  |  |  |  |  |
|  | E x P | 1.07 | 1 | 0.302 |  |  |  |  |  |  |
|  | T x P | **5.10** | **1** | **0.024** |  |  |  |  |  |  |
|  | A x E x T | 0.93 | 1 | 0.336 | 0.35 | 1 | 0.554 | 3.03 | 1 | 0.082 |
|  | A x E x P | 0.25 | 1 | 0.615 |  |  |  |  |  |  |
|  | A x T x P | 0.36 | 1 | 0.549 |  |  |  |  |  |  |
|  | E x T x P | 0.05 | 1 | 0.829 |  |  |  |  |  |  |
|  | A x E x T x P | 2.68 | 1 | 0.101 |  |  |  |  |  |  |
| Benzyl nitrile | Trait (A) | **13.63** | **1** | **0.000** | **13.92** | **1** | **<0.001** | 2.02 | 1 | 0.155 |
|  | Temperature environment (E) | **5.26** | **1** | **0.022** | **4.85** | **1** | **0.028** | 0.92 | 1 | 0.339 |
|  | Temperature genotype (T) | 0.02 | 1 | 0.897 | 1.88 | 1 | 0.171 | 2.35 | 1 | 0.125 |
|  | Pollination genotype (P) | **12.96** | **1** | **0.000** |  |  |  |  |  |  |
|  | A x E | 0.78 | 1 | 0.377 | 0.34 | 1 | 0.559 | 0.39 | 1 | 0.533 |
|  | A x T | 0.51 | 1 | 0.475 | 0.03 | 1 | 0.873 | 0.92 | 1 | 0.337 |
|  | E x T | 3.09 | 1 | 0.079 | 2.47 | 1 | 0.116 | 0.74 | 1 | 0.390 |
|  | A x P | **5.00** | **1** | **0.025** |  |  |  |  |  |  |
|  | E x P | 1.47 | 1 | 0.226 |  |  |  |  |  |  |
|  | T x P | **4.33** | **1** | **0.037** |  |  |  |  |  |  |
|  | A x E x T | 3.09 | 1 | 0.079 | 3.49 | 1 | 0.062 | 0.51 | 1 | 0.473 |
|  | A x E x P | 0.13 | 1 | 0.718 |  |  |  |  |  |  |
|  | A x T x P | 0.79 | 1 | 0.374 |  |  |  |  |  |  |
|  | E x T x P | 0.39 | 1 | 0.530 |  |  |  |  |  |  |
|  | A x E x T x P | 0.99 | 1 | 0.320 |  |  |  |  |  |  |
| Methyl salicilate | Trait (A) | **5.59** | **1** | **0.018** | **5.95** | **1** | **0.015** | 0.63 | 1 | 0.429 |
|  | Temperature environment (E) | 2.33 | 1 | 0.127 | 2.10 | 1 | 0.147 | 0.47 | 1 | 0.494 |
|  | Temperature genotype (T) | 0.00 | 1 | 0.985 | 1.16 | 1 | 0.281 | 1.23 | 1 | 0.268 |
|  | Pollination genotype (P) | **17.22** | **1** | **0.000** |  |  |  |  |  |  |
|  | A x E | 0.05 | 1 | 0.825 | 0.35 | 1 | 0.553 | 0.07 | 1 | 0.788 |
|  | A x T | 2.25 | 1 | 0.133 | **7.35** | **1** | **0.007** | 0.95 | 1 | 0.329 |
|  | E x T | 3.33 | 1 | 0.068 | **4.84** | **1** | **0.028** | 0.13 | 1 | 0.716 |
|  | A x P | 1.39 | 1 | 0.238 |  |  |  |  |  |  |
|  | E x P | 0.45 | 1 | 0.501 |  |  |  |  |  |  |
|  | T x P | 2.32 | 1 | 0.128 |  |  |  |  |  |  |
|  | A x E x T | 0.05 | 1 | 0.826 | 1.47 | 1 | 0.226 | 1.08 | 1 | 0.298 |
|  | A x E x P | 0.33 | 1 | 0.568 |  |  |  |  |  |  |
|  | A x T x P | **6.90** | **1** | **0.009** |  |  |  |  |  |  |
|  | E x T x P | 2.00 | 1 | 0.157 |  |  |  |  |  |  |
|  | A x E x T x P | 2.67 | 1 | 0.102 |  |  |  |  |  |  |
| Indole | Trait (A) | **10.48** | **1** | **0.001** | **7.26** | **1** | **0.007** | 2.93 | 1 | 0.087 |
|  | Temperature environment (E) | 2.39 | 1 | 0.122 | 1.73 | 1 | 0.189 | 0.63 | 1 | 0.428 |
|  | Temperature genotype (T) | 0.04 | 1 | 0.846 | 1.75 | 1 | 0.186 | 2.65 | 1 | 0.103 |
|  | Pollination genotype (P) | **13.73** | **1** | **0.000** |  |  |  |  |  |  |
|  | A x E | 0.04 | 1 | 0.841 | 0.44 | 1 | 0.508 | 0.09 | 1 | 0.765 |
|  | A x T | 0.66 | 1 | 0.416 | 0.25 | 1 | 0.616 | 1.82 | 1 | 0.177 |
|  | E x T | 3.03 | 1 | 0.082 | 2.59 | 1 | 0.108 | 0.62 | 1 | 0.431 |
|  | A x P | 2.10 | 1 | 0.148 |  |  |  |  |  |  |
|  | E x P | 0.50 | 1 | 0.480 |  |  |  |  |  |  |
|  | T x P | **4.63** | **1** | **0.031** |  |  |  |  |  |  |
|  | A x E x T | 3.10 | 1 | 0.078 | **6.92** | **1** | **0.009** | 0.00 | 1 | 0.992 |
|  | A x E x P | 0.44 | 1 | 0.505 |  |  |  |  |  |  |
|  | A x T x P | 2.29 | 1 | 0.130 |  |  |  |  |  |  |
|  | E x T x P | 0.55 | 1 | 0.460 |  |  |  |  |  |  |
|  | A x E x T x P | **4.31** | **1** | **0.038** |  |  |  |  |  |  |
| *(E,E)*-α-Farnesene | Trait (A) | **6.99** | **1** | **0.008** | 1.68 | 1 | 0.195 | **4.49** | **1** | **0.034** |
|  | Temperature environment (E) | 2.13 | 1 | 0.144 | 1.06 | 1 | 0.302 | 0.89 | 1 | 0.347 |
|  | Temperature genotype (T) | 0.30 | 1 | 0.581 | 1.20 | 1 | 0.273 | 3.55 | 1 | 0.060 |
|  | Pollination genotype (P) | **14.30** | **1** | **0.000** |  |  |  |  |  |  |
|  | A x E | 0.52 | 1 | 0.469 | 0.01 | 1 | 0.938 | 1.12 | 1 | 0.290 |
|  | A x T | 0.03 | 1 | 0.865 | 0.38 | 1 | 0.539 | 0.93 | 1 | 0.334 |
|  | E x T | 1.67 | 1 | 0.196 | 1.91 | 1 | 0.167 | 0.14 | 1 | 0.707 |
|  | A x P | 0.03 | 1 | 0.862 |  |  |  |  |  |  |
|  | E x P | 0.18 | 1 | 0.673 |  |  |  |  |  |  |
|  | T x P | **4.39** | **1** | **0.036** |  |  |  |  |  |  |
|  | A x E x T | 2.68 | 1 | 0.101 | **5.90** | **1** | **0.015** | 0.06 | 1 | 0.802 |
|  | A x E x P | 0.35 | 1 | 0.557 |  |  |  |  |  |  |
|  | A x T x P | 1.83 | 1 | 0.176 |  |  |  |  |  |  |
|  | E x T x P | 0.68 | 1 | 0.409 |  |  |  |  |  |  |
|  | A x E x T x P | **4.06** | **1** | **0.044** |  |  |  |  |  |  |
| Total volatile emission | Trait (A) | **10.16** | **1** | **0.001** | **6.34** | **1** | **0.012** | 2.84 | 1 | 0.092 |
|  | Temperature environment (E) | 2.33 | 1 | 0.127 | 1.84 | 1 | 0.175 | 0.56 | 1 | 0.455 |
|  | Temperature genotype (T) | 0.24 | 1 | 0.625 | 0.98 | 1 | 0.323 | 2.75 | 1 | 0.097 |
|  | Pollination genotype (P) | **11.58** | **1** | **0.001** |  |  |  |  |  |  |
|  | A x E | 0.40 | 1 | 0.529 | 0.74 | 1 | 0.389 | 0.00 | 1 | 0.974 |
|  | A x T | 0.73 | 1 | 0.394 | 0.03 | 1 | 0.874 | 1.69 | 1 | 0.194 |
|  | E x T | 3.35 | 1 | 0.067 | 3.31 | 1 | 0.069 | 0.49 | 1 | 0.484 |
|  | A x P | 1.51 | 1 | 0.220 |  |  |  |  |  |  |
|  | E x P | 0.51 | 1 | 0.474 |  |  |  |  |  |  |
|  | T x P | 3.69 | 1 | 0.055 |  |  |  |  |  |  |
|  | A x E x T | **4.88** | **1** | **0.027** | **9.85** | **1** | **0.002** | 0.00 | 1 | 0.996 |
|  | A x E x P | 0.41 | 1 | 0.523 |  |  |  |  |  |  |
|  | A x T x P | 1.45 | 1 | 0.228 |  |  |  |  |  |  |
|  | E x T x P | 1.26 | 1 | 0.261 |  |  |  |  |  |  |
|  | A x E x T x P | **5.97** | **1** | **0.015** |  |  |  |  |  |  |
| Sum of aromatic compounds | Trait (A) | **14.46** | **1** | **0.000** | **15.32** | **1** | **<0.001** | 2.13 | 1 | 0.144 |
|  | Temperature environment (E) | 3.56 | 1 | 0.059 | 3.38 | 1 | 0.066 | 0.63 | 1 | 0.426 |
|  | Temperature genotype (T) | 0.05 | 1 | 0.825 | 1.47 | 1 | 0.225 | 2.24 | 1 | 0.135 |
|  | Pollination genotype (P) | **11.56** | **1** | **0.001** |  |  |  |  |  |  |
|  | A x E | 0.09 | 1 | 0.770 | 0.39 | 1 | 0.534 | 0.01 | 1 | 0.909 |
|  | A x T | 1.01 | 1 | 0.315 | 0.06 | 1 | 0.811 | 2.00 | 1 | 0.157 |
|  | E x T | 3.55 | 1 | 0.059 | 3.17 | 1 | 0.075 | 0.71 | 1 | 0.398 |
|  | A x P | **5.61** | **1** | **0.018** |  |  |  |  |  |  |
|  | E x P | 0.97 | 1 | 0.324 |  |  |  |  |  |  |
|  | T x P | **4.06** | **1** | **0.044** |  |  |  |  |  |  |
|  | A x E x T | 3.67 | 1 | 0.056 | **6.47** | **1** | **0.011** | 0.12 | 1 | 0.726 |
|  | A x E x P | 0.31 | 1 | 0.575 |  |  |  |  |  |  |
|  | A x T x P | 1.45 | 1 | 0.229 |  |  |  |  |  |  |
|  | E x T x P | 0.85 | 1 | 0.355 |  |  |  |  |  |  |
|  | A x E x T x P | 3.29 | 1 | 0.070 |  |  |  |  |  |  |

**Table S11. The evolutionary rates of plant traits.** Output of (generalized) linear mixed models showing effects of temperature- and biotic-mediated evolution on the evolutionary rates of plant visual traits, plant rewards, phenology and plant floral scent emission, assessed in Haldanes (s.d. per generation) using plants from generations 1 and 8. Bold values indicate results where *P* ≤ 0.05.

| N | Factors | χ^2^ | DF | P |
| --- | --- | --- | --- | --- |
| 368 | Temperature genotype (T) | 0.30 | 1 | 0.582 |
|  | **Pollination genotype (P)** | **15.12** | **1** | **<0.001** |
|  | Trait Group (TG) | 0.19 | 1 | 0.666 |
|  | T*P | 1.93 | 1 | 0.165 |
|  | T*TG | 0.14 | 1 | 0.707 |
|  | P*TG | 0.53 | 1 | 0.467 |
|  | T*P*TG | 0.47 | 1 | 0.494 |


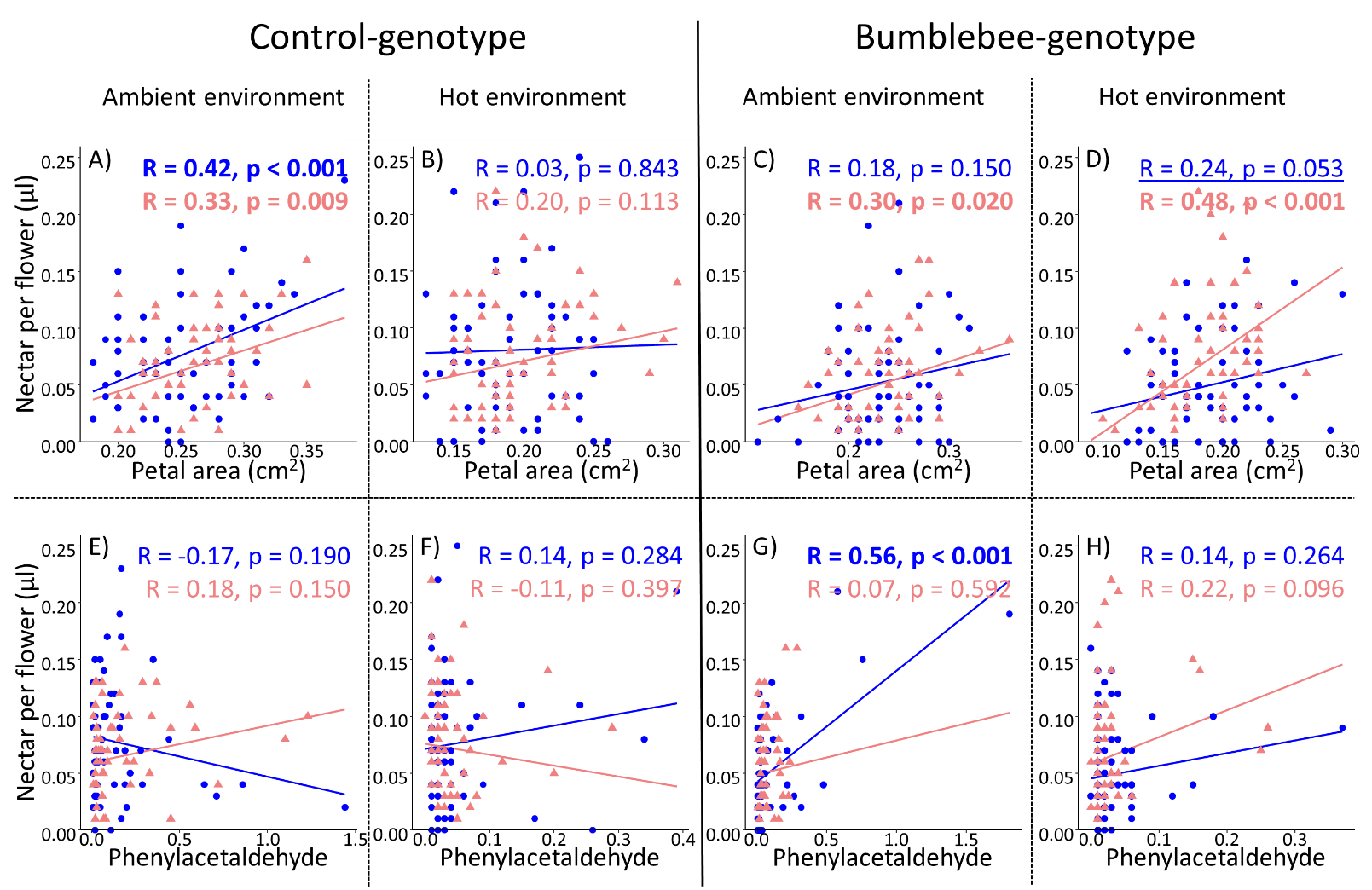


**Figure S1.** **The effect of pollinator-mediated evolution and temperature environment on the honest signalling of ambient- and hot-genotype plants.** Scatterplots and Pearson coefficients show the correlation between plant rewards (nectar per flower) and pollinator advertisement traits in ambient-genotype (blue) and hot-genotype (red) plants. The advertisement traits tested are petal area (A-D) and phenylacetaldehyde emission (E-H). Scent emission is ln+1 transformed and expressed as pg / flower / l / h. Scatterplots and correlations were performed separately for each pollination genotype and temperature environment. Bold values indicate results where *P* ≤ 0.05. Underlined values indicate results where *P* ≤ 0.07.
